# Forage lignocellulose is an important factor in driving the seasonal dynamics of rumen anaerobic fungi in grazing yak and cattle

**DOI:** 10.1128/spectrum.00788-23

**Published:** 2023-09-14

**Authors:** Zeyi Liang, Jianbo Zhang, Anum Ali Ahmad, Jianlin Han, Javad Gharechahi, Mei Du, Juanshan Zheng, Peng Wang, Ping Yan, Ghasem Hosseini Salekdeh, Xuezhi Ding

**Affiliations:** 1 Key Laboratory of Yak Breeding Engineering, Lanzhou Institute of Husbandry and Pharmaceutical Sciences, Chinese Academy of Agricultural Sciences, Lanzhou, China; 2 Key Laboratory of Veterinary Pharmaceutical Development, Ministry of Agricultural and Rural Affairs, Lanzhou Institute of Husbandry and Pharmaceutical Sciences, Chinese Academy of Agricultural Sciences, Lanzhou, China; 3 Livestock Genetics Program, International Livestock Research Institute (ILRI), Nairobi, Kenya; 4 CAAS-ILRI Joint Laboratory on Livestock and Forage Genetic Resources, Institute of Animal Science, Chinese Academy of Agricultural Sciences (CAAS), Beijing, China; 5 Human Genetics Research Center, Baqiyatallah University of Medical Sciences, Tehran, Iran; 6 Department of Systems Biology, Agricultural Biotechnology Research Institute of Iran, Agricultural Research, Education, and Extension Organization, Karaj, Iran; 7 Department of Molecular Sciences, Macquarie University, North Ryde, New South Wales, Australia; Oklahoma State University, Stillwater, Oklahoma, USA

**Keywords:** yak, cattle, natural grazing, rumen anaerobic fungi, seasonal dynamics

## Abstract

Anaerobic fungi (AF) inhabit the gastrointestinal tract of ruminants and play an important role in the degradation of fiber feed. However, limited knowledge is available on seasonal dynamics and inter-species differences in rumen AF community in yak and cattle under natural grazing systems. Using the random forests model, the null model, and structural equation model, we investigated the seasonal dynamics and key driving factors of fiber-associated rumen AF in grazing yak and cattle throughout the year on the Qinghai-Tibet Plateau (QTP). We found that the richness and diversity of rumen AF of grazing yak and cattle in cold season were significantly higher than those in warm season (*P* < 0.05). We identified 12 rumen AF genera, among which , *Cyllamyces*, and *Orpinomyces* were predominant in the rumen of both grazing yak and cattle. LEfSe and random forest analysis showed that *Feramyces*, *Tahromyces*, and *Buwchfawromyces* were important seasonal indicator of rumen AF in grazing yak (*P* < 0.05), and *Caecomyces*, *Cyllamyces*, and *Piromyces* in grazing cattle (*P* < 0.05). Null model analysis revealed that the dynamic changes of rumen AF community structure were mainly affected by deterministic factors. Notably, mantel test and structural equation model revealed that forage physical-chemical properties, including dry matter (DM), neutral detergent fiber (NDF), and hemicellulose contents (HC) were the key factors driving the seasonal variations of the rumen AF community (*P* < 0.05). The results revealed that forage lignocellulose was probably an important factor affecting the seasonal dynamics and inter-species differences of the rumen AF community under natural grazing conditions.

**IMPORTANCE:**

The seasonal dynamics of rumen anaerobic fungi in nature grazing yak and cattle were determined during cold and warm seasons based on pasture nutritional quality and environmental data sets. The main driving factors of anaerobic fungi in yak and cattle rumen were explored by combining random forest and structural equation models. In addition, the dynamic differences in the composition of the anaerobic fungi community in the yak and cattle in different seasons were characterized. It was found that some rumen anaerobic fungi have contributed to high fiber degradation rate in yak. These novel findings improve our understanding of the association of environmental and dietary seasonal variations with anaerobic fungal community, facilitating yak adaptation to high altitude.

## INTRODUCTION

With the rapid growth of the world’s human population, demand for foods is sharply increasing, especially for animal-sourced proteins (1). Ruminants can use their rumen microecological system to ferment and utilize plant cellulose fibrils into high-quality animal proteins (e.g., meat and milk) for human consumption (2, 3). It is well known that the gastrointestinal tracts of herbivores, especially the rumen of ruminants, represent one of nature’s most highly evolved and efficient plant biomass-degrading ecosystems (2 – 6). Previous studies have also reported that a large number of symbiotic microbial communities (bacteria, archaea, fungi, etc.) in the rumen can effectively degrade complex lignocellulosic biomasses and non-protein nitrogen from crop residues into volatile fatty acids (VFAs), microbial proteins, and vitamins to provide nutrients to their hosts for growth and production (2, 3, 5, 6).

The yak, a large herbivore species exclusively inhabiting the QTP and adjacent mountainous regions, evolutionarily diverged from cattle around 4.4 to 5.3 million years ago (7). To date, yak are still raised mainly by nature grazing, with coarse grass as their only feed source (8, 9). In long-term evolution, yak have developed a unique rumen microbiota with strong fiber degradation ability, which can resist extreme environments and imbalances in seasonal feed and nutrition supply (10). Several studies have shown that yak can more effectively degrade lignocellulose compared to cattle under the same conditions, by providing their body with more energy and nitrogen (10, 11). In the long cold season on the QTP (November to May of the next year), the quantity and quality of natural forage cannot meet the nutritional needs of yak (8), but they can still survive through the long winter due probably to their efficient fiber degradation ability by rumen microorganisms.

Rumen AF are known to a play key role in the degradation of plant lignocellulosic materials (12, 13), although the rRNA transcript abundances indicate that AF represent 10% to 20% of the rumen microbiota (14, 15), suggesting that abundance of rumen AF is relatively low compared with bacteria and archaea. However, rumen anaerobic fungi are considered to play key roles in the degradation of plant lignocellulosic materials (12, 13). Rumen AF can produce high cellulolytic and xylanolytic enzymes, which penetrate and break fiber feed particles, thus weakening the integrity of feed fiber. Therefore, rumen AF are considered as the primary invaders to initiate the degradation of fiber feed particles (16 – 18). In an early study, it was found that the digestibility of dry matter in the rumen of sheep with fungi increased by 14% to 40% compared to the sheep without fungi (19). Additionally, *in vitro* studies have also shown that the contribution of AF to ruminal degradation of plant substrates could be more significant than rumen cellulolytic bacteria (12, 20). Elimination of AF from sheep rumen decreased straw digestion up to 9.2%, which was reversed when AF were reintroduced (21). Recently, several “omics” studies demonstrated that the differences in rumen microbiota are associated with cattle health and production (22 – 24), including ruminal acidosis, feed efficiency and milk component. Although diet has a significant effect on the structure of rumen AF community, recent studies have shown that there are still many other factors influencing rumen microbiota, including host genetic background, feeding frequency, dry matter intake and grazing season (6, 25 – 27). There is limited knowledge about seasonal changes and differences in the rumen AF community in natural grazing yak and cattle living on the QTP, as well as the driving factors of dynamics of the rumen AF community and the response patterns of their hosts.

In recent years, many studies have made significant efforts in understanding how rumen bacteria and archaea affect the nutrition, resilience and environmental footprint of ruminants (14, 28, 29). As far as we know, there are relatively few reports on the dynamic changes of rumen AF and its impact on host animal species. However, techniques for exploring gut fungi, such as 18S ribosomal DNA and internal transcribed spacers (ITS, 1 and 2), are still limited compared to gut bacteria and archaea (30, 31). Unfortunately, the ITS region was amplified in this study, which might have resulted in a limited number of annotations of anaerobic fungal species. For future use, it is recommended to use the primers designed for the D1–D2 region of the large ribosomal subunit of AF (32), to evaluate the community structure and function of rumen AF in yak. A better assessment of the differences in AF community compositions between grazing yak and cattle rumen ecosystems will help us better understand how they adapt to extreme environments on the QTP. The purpose of this study was to evaluate ruminal AF community dynamics and important driving factors in yak and cattle during different grazing months. Rumen fluid and blood samples from yak (*n* = 9) and cattle (*n* = 9) naturally grazed on the same pasture every other month were collected, as well as the environmental factors such as monthly average temperature and forage nutrient quality around the pasture were recorded. The rumen AF dynamics of adult grazing yak and cattle were studied by using the ITS1 amplification sequencing technology. Combined with temperature, humidity and forage nutrients data, the main factors driving AF seasonal changes were explored. We identified the anaerobic-specific fungal community in yak rumen and revealed the microbial mechanism of host adaptation to harsh alpine environments.

## RESULTS

### Variations in environmental and host factors in different grazing months

We found that the climatic variables around the pasture showed dynamic changes in different grazing months, including monthly average temperature and relative humidity (Fig. S1A through C). The average monthly temperature (including day and night) and humidity around the pasture were the lowest in January and the highest in July according to the data released by the Gansu Meteorological Station. There were significant differences in forage nutrients between different grazing months (*P* < 0.05). The content of forage biome (BM), crude protein (CP) and other extract (EE) gradually increased from November to July and then gradually decreased until September. Other forage nutrient indexes showed a gradual decrease from November to May, followed by gradual increases till September (Fig. S1D through O). All rumen VFA concentrations of yak and cattle were significantly different across the grazing months (*P* < 0.001), with significantly higher concentrations in the warm season (*P* < 0.05; Table S1). In addition, we found that the concentrations of rumen isobutyric acid (IBUT) and isovaleric acid (IVAL) were significantly higher in the rumen of yak than cattle (*P* < 0.001; Table S1). The results of blood biochemical indicators showed that glucose (GLU), low-density lipoprotein cholesterol (LDL-C), total cholesterol (TC) and non-esterified fatty acid (NEFA) were significantly lower in yak than cattle (*P* < 0.01), and LDH, high-density lipoprotein cholesterol (HDL-C) and TC were significantly higher in the warm season compared to cold season (*P* < 0.01; Table S1).

### Diversity of the rumen AF community during different grazing months

**Fig 1:**
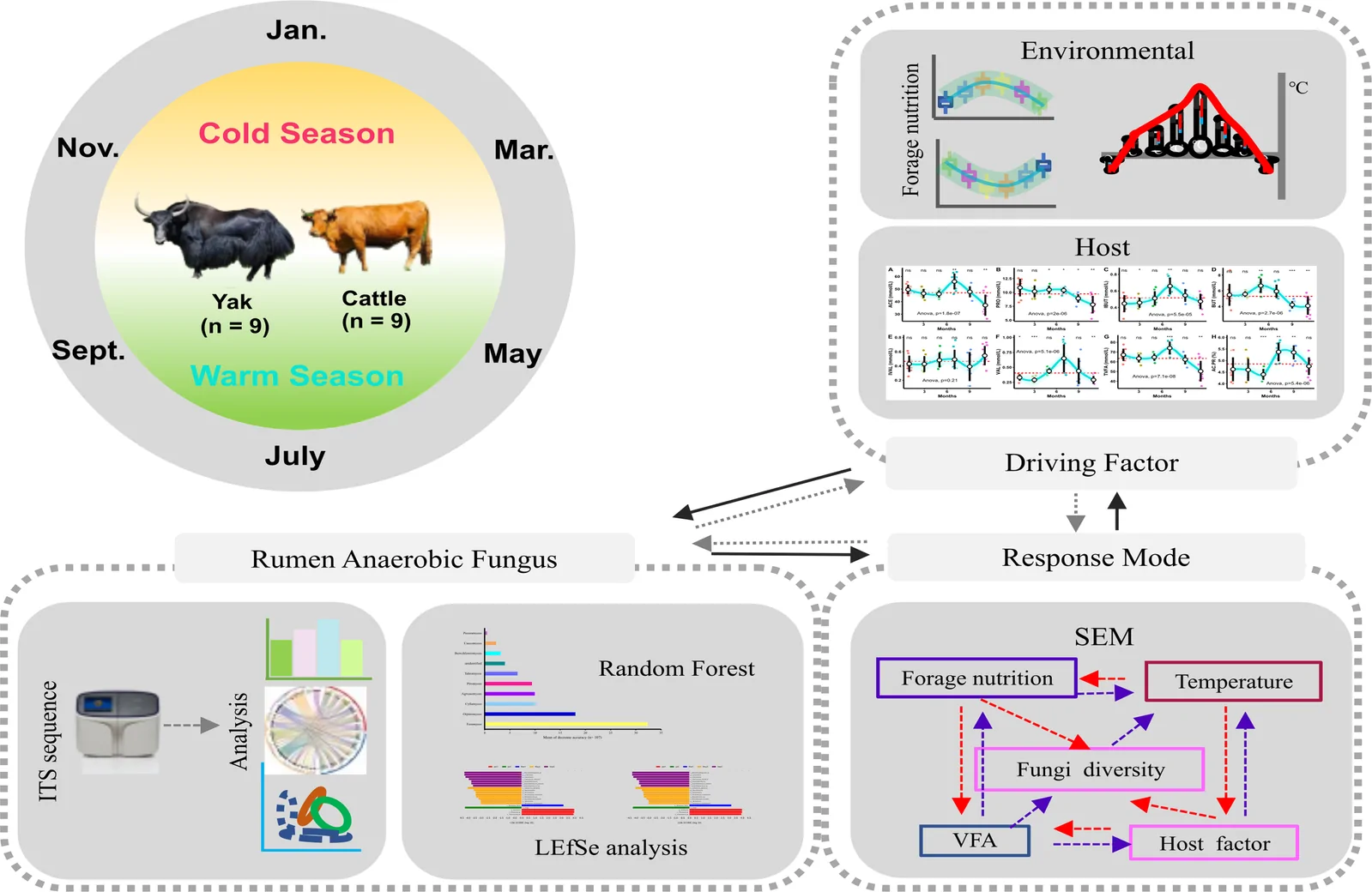
Schematic of the experimental design.

To reveal the dynamics and driving factors of the rumen AF community during different grazing months, the ITS-based amplicon sequencing was performed (Fig. 1). Across all the samples (*n* = 108), we obtained a total of 8,342,004 high-quality fungal sequences, which were grouped into 13,407 OTUs using 97% sequence similarity cutoff value. The result of this study indicated that the dominant fungi at the phylum level of cattle and yak were Ascomycota, Neocallimastigomycota, and Basidiomycota (Fig. S2A through C), to be consistent with previous finding (33). Early studies have reported that Neocollimastigomycota inhabits the rumen and digestive tract of herbivores and might play an important role in the degradation of plant fibers (34, 35). By comparing the UNITE database and combining the sequences deposited at the NCBI databases, we obtained 545 OTUs belonging to the phylum Neocallimastigomycota. In the Neocollimatigomycota phylum, the first three genera to be dominant in cattle and yak were *Caecomycos*, *Cylamyces*, and *Pyromyces* (Fig. S2B and D).

**Fig 2:**
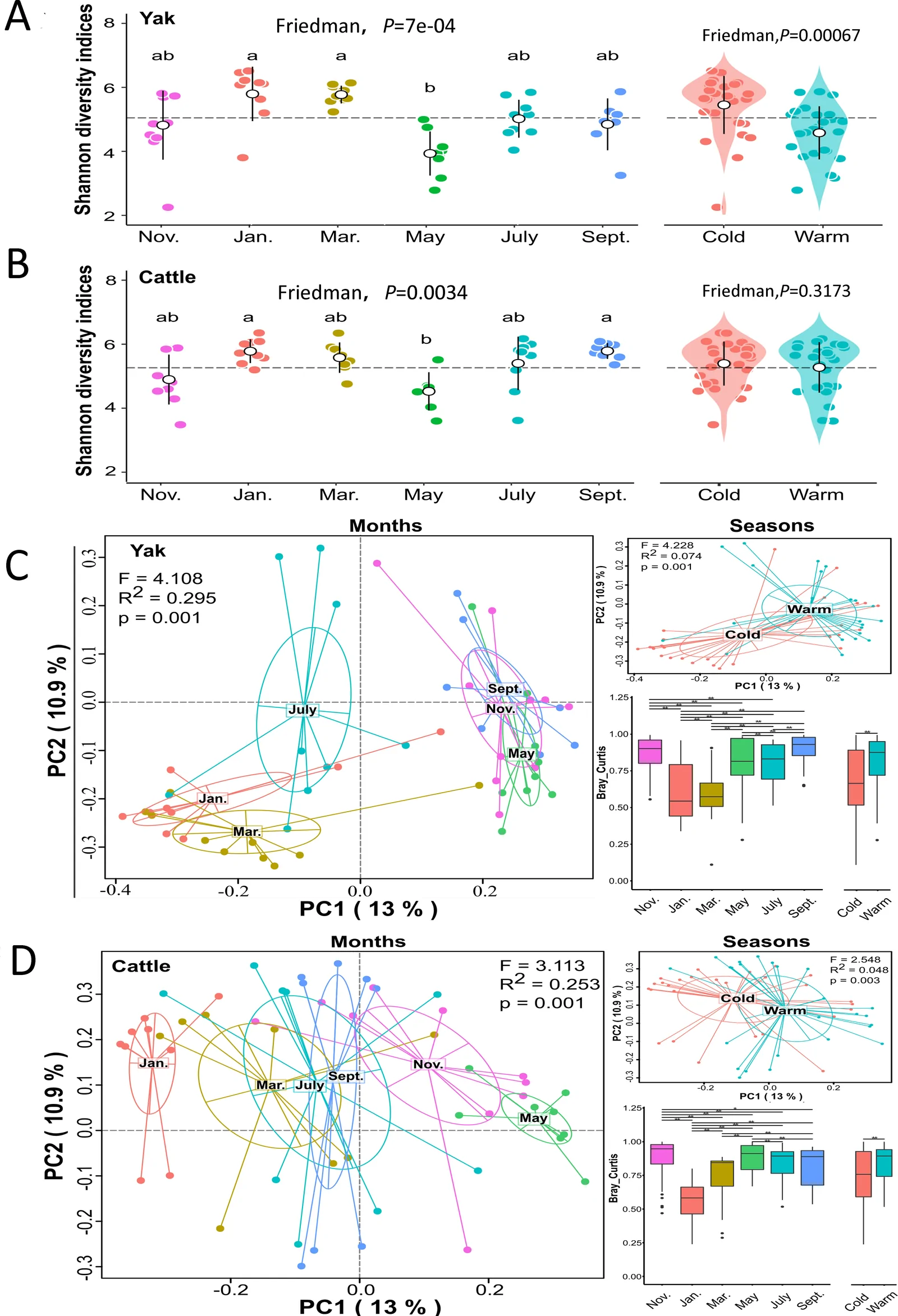
Effects of different grazing months and seasons on the diversity and structure of rumen anaerobic fungi community of yak and cattle. Scatter plots show that the diversity indices of rumen anaerobic fungi community of yak (**A**) and cattle (**B**) during different grazing months (Nov., Jan., Mar., May, July, and Sept.) and seasons (cold and warm seasons). The Friedman non-parametric test was used to compare the diversity indices between different months and between different seasons. PCoA plots based on Bray-Curtis distances matrix show the effects of different grazing months and seasons on rumen anaerobic fungi community structures in yak (**C**) and cattle (**D**). The effects of different grazing months and seasons on the anaerobic fungi structures were compared by the PERMANOVA.

The alpha diversity indices of the rumen AF community in yak and cattle increased from November to March and from May to September, then decreased from March to May (Fig. 2A and B; Fig. S3A and B). The Chao1 and Shannon diversity indices of the yak rumen AF community were significantly higher in the cold season than warm season (Friedman test, Chao1, *P* = 0.0093; Shannon, *P* = 0.00067; Fig. 2A; Fig. S3A). We found that the value of the Chao1 index in cattle was significantly higher in the cold season than warm season (Friedman test, Chao1, *P* = 0.003; Fig. S3B), but there was no significant difference in the Shannon index (Friedman test, Shannon, *P* = 0.3173; Fig. 2B). However, we found that the Shannon index and Chao1 index of rumen AF were significantly higher in cattle than yak in September (Friedman test, Chao1, *P* < 0.001; Shannon, *P* < 0.001; Fig. S3C and D), while there was no significant difference in other months. Beta diversity analysis revealed significant changes in different grazing months and seasons (Fig. 2C and D; Fig. S3E). Except in July, there were significant differences in the community structures of rumen AF between yak and cattle in the other five grazing months (*P* < 0.05) (Table S2; Fig. S3E). No significant difference was observed in yak rumen AF community structures between September and November (PERMANOVA, F_1,17_ = 1.219, *P* = 0.169), but significant differences were observed between other grazing months (PERMANOVA, *P* < 0.01; Fig. 2C; Table S2). For cattle, the structure of the rumen AF community in July was similar to than in March (JulyC vs. MarC: F_1,15_ = 1.677, *P* = 0.075) and September (JulyC vs. SeptC: F_1,15_ = 1.488, *P* = 0.113), but significant differences were observed between other grazing months (Fig. 2D; Table S2). Overall, our data suggested that the rumen AF community of grazing yak and cattle might be affected by grazing months; though we realized that the D1–D2 region must be explored to further confirm these results.

### The composition of the rumen AF community was different between grazing yak and cattle

**Fig 3:**
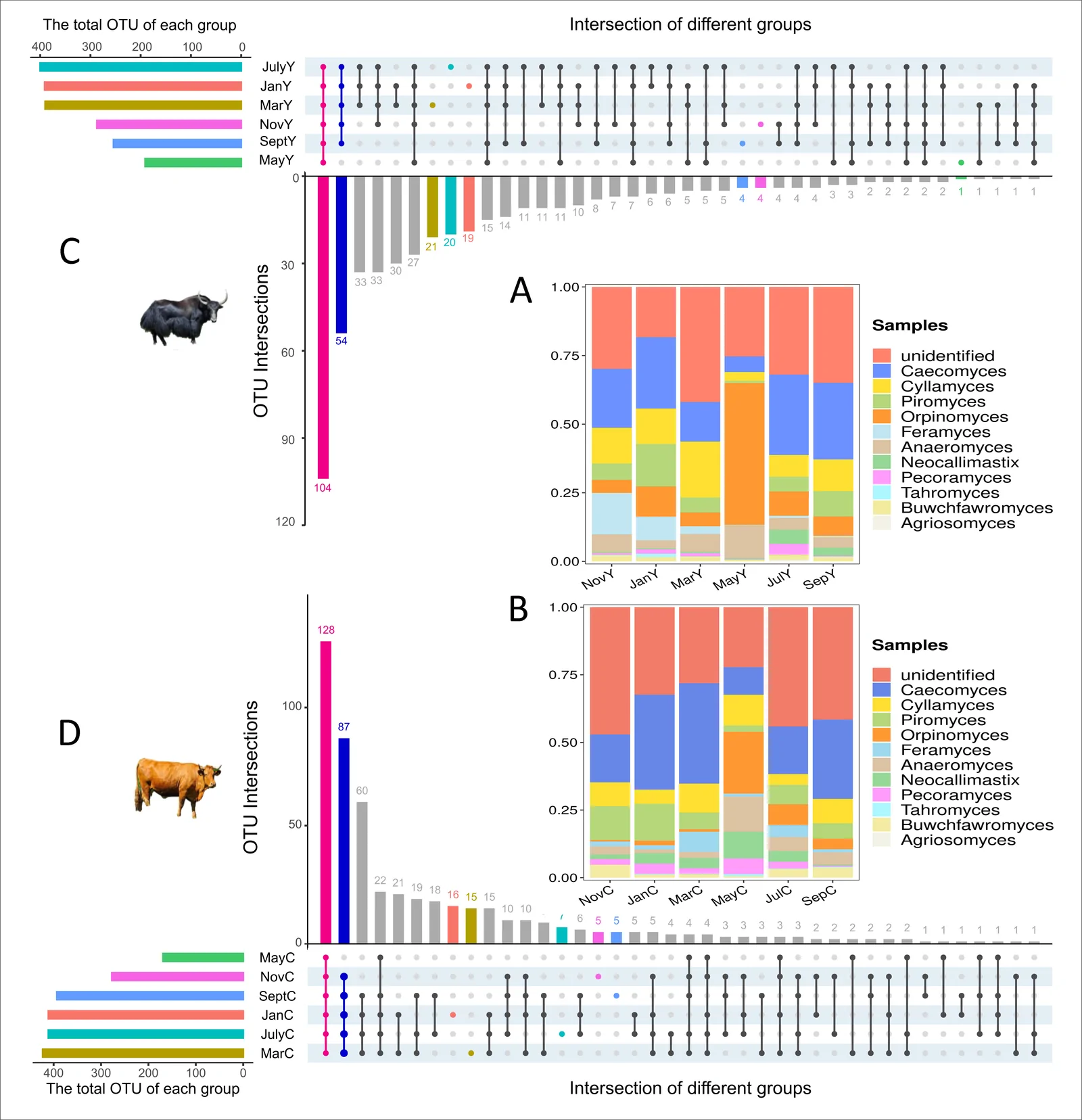
Composition of rumen anaerobic fungi in yak and cattle during different grazing months. (**A**) and (**B**) Histograms show the distribution of rumen anaerobic fungi sequences in yak and cattle in different grazing months, respectively. Yak and cattle are displayed separately. Upset plots show the distribution of the rumen anaerobic fungi OTUs in yak (**C**) and cattle (**D**) sampled at different grazing months (Nov., Jan., Mar., May, July and Sept.).

We observed 12 genera in rumen AF (Fig. 3A and B; Fig. S4A), of which *Caecomyces* (Y: 23.07%; C: 30.22%), *Cyllamyces* (Y: 13.27%; C: 6.80%), *Orpinomyces* (Y: 10.04%; C: 3.12%), and *Piromyces* (Y: 9.34%; C: 9.32%) were predominant in the rumen of yak and cattle. The remaining sequences were affiliated with *Anaeromyces* (Y: 4.56%; C: 3.25%), *Feramyces* (Y: 4.10%; C: 3.25%), *Pecoramyces* (Y: 2.19%; C: 2.42%), *Neocallimastix* (Y: 1.88%; C: 3.52%), *Buwchfawromyces* (Y: 1.66%; C: 2.10%), *Tahromyces* (Y: 0.54%; C: 0.19%), *Agriosomyces* (Y: 0.23%; C: 0.04%) and *unidentified Neocallimastigaceae* (Y:29.12%; C: 36.12%). Sixty-three OTUs (11.56%) were shared between yak and cattle throughout the grazing months, including the members of *Caecomyces*, *Piromyces,* and *Cyllamyces* dominant in the rumen AF community (Fig. S4A). Among shared AF communities, members of the genera *Cyllamyces*, *Orpinomyces,* and *Anaeromyces* were over represented in yak compared to cattle. In addition, the OTUs shared by yak and cattle during different grazing months were 104 and 128, respectively, which mainly included the members of *Caecomyces*, *Piromyces*, *Cyllamyces*, and *Orpinomyces* (Fig. 3C and D; Fig. S4B and C).

**Fig 4:**
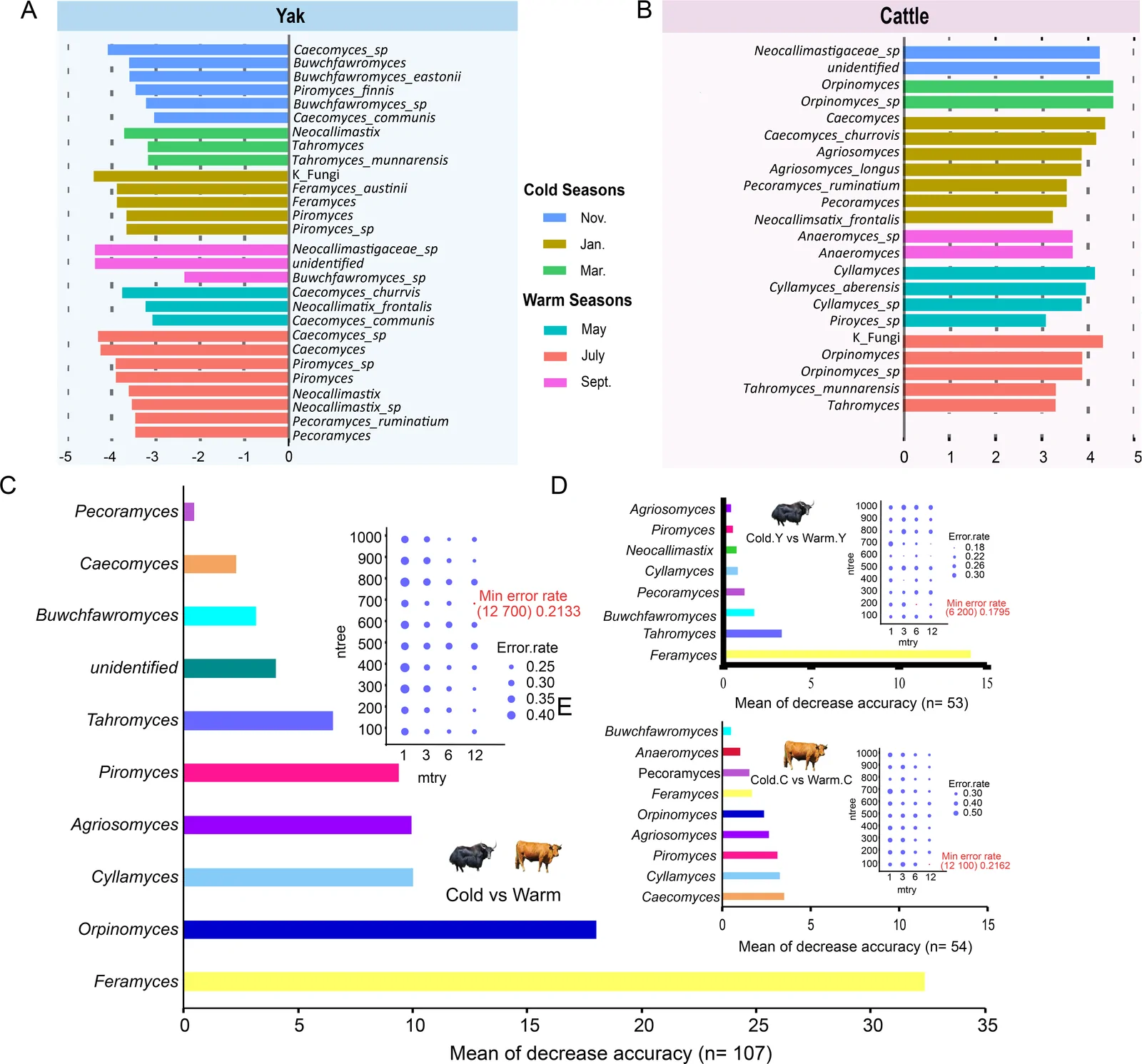
Dominant taxa differentiating the rumen anaerobic fungi community of yak and cattle during seasonal changes in diet and climate. The relative importance of rumen anaerobic fungi community of yak (**A**) and cattle (**B**) in cold season (Nov., Jan., and Mar.) and warm season (May, July, and Sept.) were determined by the LEfSe. The bar chart shows the anaerobic fungi with significant differences in the rumen of yak and cattle in different grazing months. Different colors represent different grazing months. (**C**) The relative abundances of rumen anaerobic fungi community in yak and cattle were classified at genus level in cold and warm seasons to identify top 10 anaerobic fungi as important biomarker taxa based on their effects on the accuracy of the model. The inserted bubble plot represents a 10-fold cross-validation error rate under different combinations for screening the best classification model and determining the significant number of biomarkers. The important anaerobic fungi biomarkers of yak (**D**) and cattle (**E**) in different grazing seasons were identified by the random forest model.

### Seasonal indicators of rumen AF were different between grazing yak and cattle

LEfSe analysis was performed to determine the effects of different grazing months on rumen AF community of yak and cattle (Fig. 4A and B; Fig. S5A and B), while seasonal changes in the relative abundances of rumen AF at the genus level in yak and cattle were assessed using the random forest model to correlate rumen AF composition with grazing cycle (Fig. 4C and D and Fig. S5C through E). During the cold season, the rumen AF community of yak mainly consisted of the genera of *Feramyces*, *Piromyces*, *Buwchfawromyce*, *Neocallimastix* and *Tahromyces*; while the genera of *Caecomyces*, *Orpinomyces*, *Agriosomyces*, and *Pecoramyces* were predominant in cattle (Fig. 4A; Fig. S4A and B). However, the rumen AF community of yak during the warm season mainly consisted of the genera of *Caecomyces*, *Piromyces*, *Neocallimastix*, and *Pecoramyces*, whereas *Cyllamyces*, *Orpinomyces*, *Tahromyces*, and *Anaeromyces* were more common in cattle (Fig. 4B; Fig. S4A and B). From the whole rumen AF community, 10 important genera were identified by the random forest model with minimum cross-validation errors, of which the genera of *Feramyces*, *Orpinces*, *Cyllamyces*, *Agriosomyces*, and *Piromyces* were dominant (Fig. 4C). In addition, eight and nine important AF genera were identified in yak and cattle rumen in different grazing seasons, respectively, of which *Feramyces*, *Tahromyces*, and *Buwchfawermyces* were the key genera in yak, while *Caecomyces*, *Cyllamyces* and *Piromyces* were the key genera in cattle (Fig. 4D and E).

### Forage nutrition was the main factor affecting the rumen AF community in grazing yak and cattle

**Fig 5:**
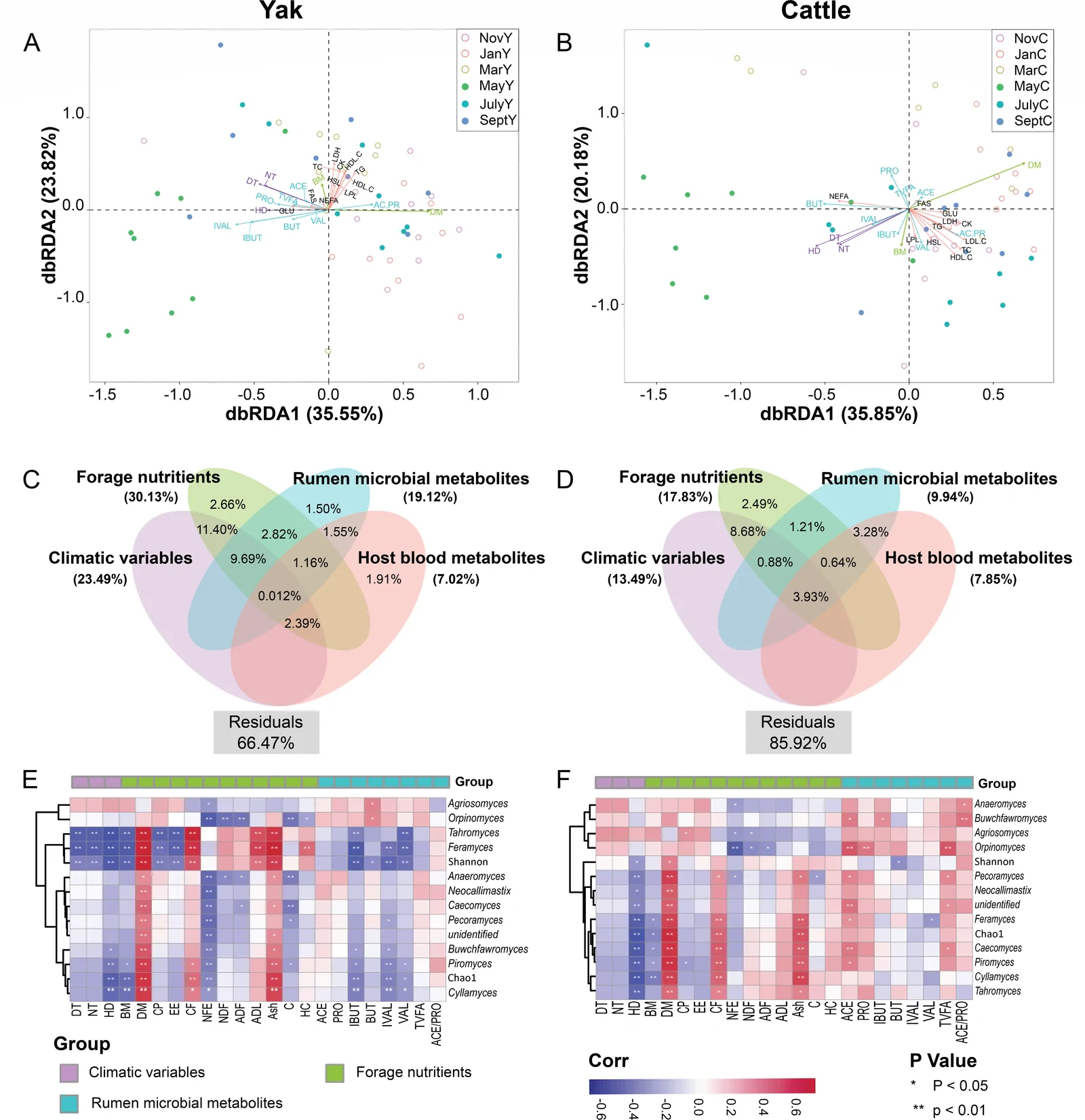
Effects of climatic variables, forage nutrients, and rumen microbial on the alteration of rumen anaerobic fungi community in yak and cattle during different grazing months and seasons. Bray-Curtis distance-based RDA (dbRDA) was used to evaluate such effects on the rumen anaerobic fungi community compositions in yak (**A**) and cattle (**B**) during different grazing months. The variances (in percentage) explained by the first two dbRDAs are shown in parentheses. Variation partitioning analysis was performed to assess the relative contributions of individual factors to the changes in rumen anaerobic fungi community in yak (**C**) and cattle (**D**). Heat maps show the influence of individual factors on the rumen anaerobic fungi at the genus level of yak (**E**) and cattle (**F**).

We evaluated the effect of climatic variables and forage nutrients during grazing months on the rumen AF community and metabolites of yak and cattle (Fig. 5; Fig. S6 and S7). The compositional variability of rumen AF community in yak and cattle can be significantly explained by climate variables, forage nutrients and rumen microbial metabolites (*P* < 0.05, permutational ANOVA for Bray Curtis distance-based redundancy analysis (dbRDA); Fig. 5A and B; Table S3). Overall, 30.13% of the variation in the rumen AF community of yak was explained by forage nutrition, while it contributed only 17.83% to the variation in the cattle rumen AF community (Fig. 5C and D). While investigating the effects of grazing months and host animal species on the rumen AF community, we found that grazing months explained 28.58% and 16.08% of the total variation in the rumen AF community of grazing yak and cattle, respectively, but the hosts explained only around 3.27% of the total variation (Fig. S8).

We further analyzed how various factors affected the rumen AF community structure of yak and cattle (Fig. 5E and F). We found that forage physical-chemical properties including DM, CF, ADL and Ash were positively correlated with *Cyllamyces*, *Feramyces*, *Tahromyces*, and *Piromyces* (*P* < 0.01, Fig. 5E and F), while NFE, ADF, and NDF were significantly and negatively correlated with *Orpinomyces* and *Anaeromyces* (*P* < 0.01, Fig. 5E). Therefore, we found that the dynamic changes of rumen AF community in grazing yak could be better explained by different forage nutrition and grazing months compared to that of cattle.

### Forage lignocellulose was the key driving factor for the dynamic change of rumen AF community

**Fig 6:**
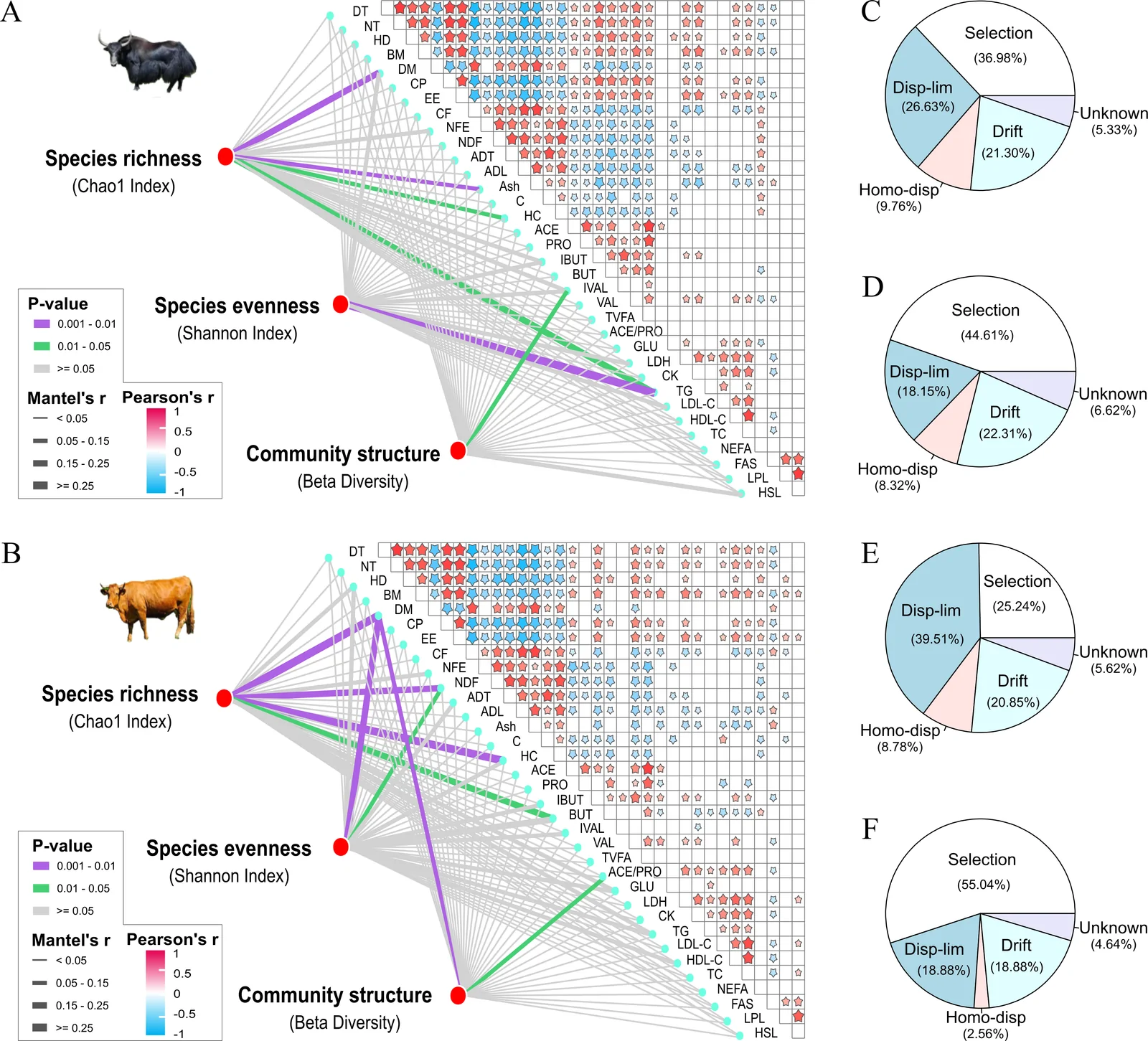
Seasonal dynamic driving factors of rumen anaerobic fungi community composition in yak and cattle. (**A**) and (**B**) Associations of rumen anaerobic fungi community composition (alpha and beta diversity) and climatic factors, forage nutrients, and rumen and blood metabolites in yak and cattle based on the Mantel test. Edge width corresponds to the Mantel’s r statistic for the corresponding distance correlations, and edge color denotes the statistical significance. The color gradients indicate the Pearson correlation coefficients between climatic and host factors, with white showing non-significant correlation at *P* = 0.05. (**C**) and (**D**) Analysis of factors affecting rumen anaerobic fungi community assembly of yak in cold and warm seasons. (**E**) and (**F**) Analysis of factors affecting rumen anaerobic fungi assembly of cattle in cold and warm seasons. The null model was used to quantify the climatic and host factors that influence the assembly of rumen anaerobic fungi community in different grazing seasons. Proportions of community pairs were assembled by drift, species sorting (selection), dispersal limitation (Disp-lim), and mass effects or homogenizing dispersal (Homo-disp).

To further investigate the roles of various deterministic factors in shaping the community structure of rumen AF, we used the partial Mantel test to correlate the differences in rumen AF community composition of yak and cattle in different grazing seasons. Among the forage nutrients, the DM and HC showed the strongest correlation with species richness of rumen AF community in both yak and cattle (*P* < 0.05), followed by ash in yak (*P* < 0.01) and NDF in cattle (*P* < 0.01). Both DM and NDF displayed correlations with species evenness, while DM was correlated with community structure in cattle (Fig. 6A and B). In addition, rumen BUT was correlated with the species richness in both cattle and yak (*P* < 0.05). Rumen IVAL and ACE/PRO had strong influences on the community structure in both yak and cattle (*P* < 0.05; Fig. 6A and B).

Since forage nutrition is a key factor affecting the composition of the rumen AF community, we hypothesized that the dynamic assembly of the rumen AF community of grazing yak and cattle had a deterministic nature. During the cold season, there was a high turnover (Yak, beta-NTI = 2.333; Cattle, beta-NTI = 1.203) of rumen AF community assembly, where environmental selection (Yak: 36.98%; Cattle: 25.24%) and dispersal limitation (Yak: 26.63%; Cattle: 39.51%) were the dominant factors in the high turnover (Fig. 6C and E; Fig. S9). In contrast, environmental selection (Yak: 44.61%; Cattle: 55.04%) was the dominant factor leading to the high turnover (Yak, beta-NTI = 2.552; Cattle, beta-NTI = 2.938) during the warm season (Fig. 6D and F; Fig. S9). These results demonstrated that the dynamic changes of rumen AF community structure were mainly affected by deterministic factors, but the key driving factors were forage physical-chemical properties, such as DM, NDF, and HC.

### Causal relationship between the environmental factors and host responses with rumen AF community

**Fig 7:**
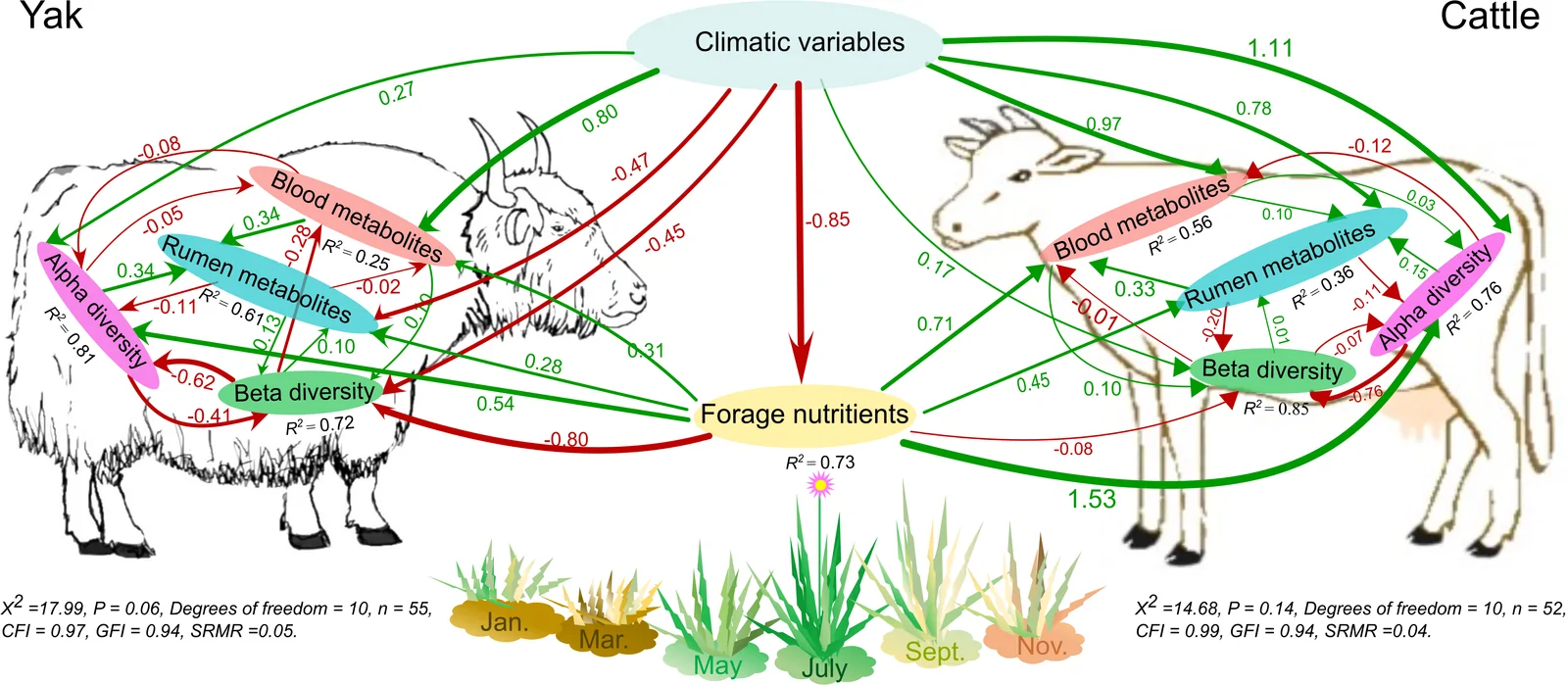
Driving factors and host response patterns of rumen anaerobic fungi community composition in yak and cattle. SEM showed the relationship between climatic variables (DT), forage nutrition (DM), and rumen (ACE/PRO) and blood (TG, LDL-C and HDL-C) metabolites and rumen anaerobic fungi community structure (alpha diversity, beta diversity) of yak and cattle. Alpha diversity is the Chao1 diversity indices based on species abundance. Beta diversity is represented by the PC1 from the Bray-Curtis distance-based PCoA. Green and red arrows represent significant (*P* < 0.05) positive and negative pathways, respectively. Numbers near the pathway arrows indicate the standard path coefficients (*β*). The arrow width is proportional to the strength of the relationship. *R^2^* represents the proportion of variance explained for every dependent variable.

Based on the above-mentioned correlations, an in-depth analysis was performed to discern the roles of individual deterministic factors using structural equation modeling (SEM). The validated SEM yielded good model fits, indicated by non-significant χ^2^ tests (*P* > 0.05); high comparative fit index (CFI >0.95); high goodness of fit index (GFI >0.95); and low standardized root mean square residual (SRMR <0.05) (36). Overall, consistent with the Mantel test result, forage nutrients had the strongest direct effects on the species richness of rumen AF (yak, standardized path coefficient: *β* = 0.54; cattle, *β* = 1.53). However, it also had negative impacts on the first principal component score (PC1) representing the community structure of rumen AF (yak, *β* = –0.80; cattle, *β* = –0.08). In grazing yak, the constructed SEM explained 81% and 72% of the total variation in species richness and community structure of rumen AF, respectively (Fig. 7). Compared to cattle (36%), the SEM better explained the rumen fermentation level of yak (61%). In grazing cattle, the constructed SEM explained 76% and 85% of the total variation in species richness and diversity of rumen AF, respectively (Fig. 7). Meanwhile, the SEM showed that there was a strong negative correlation between species richness and diversity of rumen AF of both yak and cattle, while they had weak positive correlations with rumen metabolites. Among the climatic variables, monthly mean temperatures had strong negative correlations with rumen fermentation and AF community structure of yak, indicating yak to be able to adjust the structure of rumen AF according to the changes in climatic variables for a better adaptation to the harsh natural environment on the QTP.

## DISCUSSION

In this study, we found that grazing season, forage nutritional quality, and host animal species had important effects on the seasonal dynamics of the rumen AF community, where the abundance and diversity of rumen AF in the cold season were significantly higher than those in the warm season. Additionally, we identified 11 rumen AF genera of both yak and cattle in different grazing months and also found that forage lignocellulose was probably an important factor affecting the seasonal dynamics and inter-species differences of rumen AF community under natural grazing conditions.

### Seasonal environment factors drive dynamic changes in the rumen AF community and host metabolisms

In this study, we investigated the dynamics of the rumen AF community and the factors affecting their assembly during seasonal changes in climate and forage nutrition in natural grazing yak and cattle. Our results showed that the climatic variables and forage nutrients changed in different grazing months, particularly forage DM, NDF, ADF, ADL and CF decreased gradually from January to July, but they all increased gradually from July to November (Fig. S1). Studies have shown that with the growing period, CP decreased (37) while DM, NDF, and ADF increased gradually (27, 38).

Similar to previous studies, we found that an increased forage fiber content during the cold season was associated with an increased production of ACE, resulting in a higher ACE/PRO ratio in the cold season than warm season (39). It’s worth noting that the proportions of rumen IBUT and IVAL were significantly higher in yak than in cattle during different grazing months. This may be due to the NDF digestibility and nitrogen utilization efficiency were higher in yak than in cattle (40). Some studies have found that branched-chain VFAs, such as IBUT and IVAL are essential for the growth of some cellulolytic bacteria and the digestion of structural carbohydrates in the rumen (41 – 43). Compared to other rumen microorganisms, AF is an important source of cellulases, ligninases and other hydrolase enzymes which play key roles in lignocellulose degradation in the rumen (26). In addition, we also found that the rumen metabolites of grazing yak and cattle were affected by seasonal changes in forage nutrients, and rumen metabolites such as ACE and TVAF were significantly higher in yak than cattle, consistent with the finding of Zhang et al. (10). As an important internal environment, the blood circulation system directly participates in the metabolism of substance, energy and complex biochemical processes (44). We found that blood metabolites of yak and cattle in different grazing months changed with the seasons. Certain blood metabolites, including Glu, LDH, LDL-C, and HDL-C, were significantly lower in yak compared to cattle. This might be due to the adoption of a “low-carbon” strategy of yak, invoving an improved energy efficiency, reduced energy required for maintenance, and methane emission in response to the shortage of feed during the long winter on QTP (45). A previous study reported that yak undergo the changes in the energy metabolism pathway from gluconeogenesis to glycolysis to obtain more ATP for their adaptation to the environment on the QTP (46, 47). Additionally, there were reports that yak muscles at high altitudes may have higher glucose uptake and glycogen synthesis ability (46, 48). Genetic studies on yak also showed that the rumen epithelium was enriched with genes encoding for fatty acid biosynthesis and metabolism (*Hsd17b12*) (49). We speculate that the dynamic changes in the metabolism of rumen microbiota and the in host might be caused by the changes in forage nutritional quality associated with climatic factors.

### Host animal species plays an important role in the dynamics of the rumen AF community

Although diet structure has a significant effect on the AF community composition (26, 30), host phylogeny and genetics are important factors (50 – 52). Our results showed that grazing season, forage nutrition quality, and host animal species had important effects on the dynamics of the rumen AF community (Fig. 2 and 5; Fig. S3). This is similar with some previous studies (25, 29, 52). We have annotated 12 AF genera of the Neocallimastigomycota from the rumen of yak and cattle; among them, *Caecomyces*, *Cyllamyces* and *Orpinomyces* were predominant (Fig. 4 and 5). To date, only 20 genera have been described, all belonging to Neocallimatigomycota (53). Kumar et al. (54) observed three genera of *Caecomyces*, *Cyllamyces* and *Orpinomyces* in the rumen of dairy cows, and found that their abundance were influenced by diet. In addition, both the LEfSe and random forest model analyses showed that *Feramyces*, *Tahromyces* and *Buwchfawromyces* were important AF in the rumen of yak during different grazing seasons, while *Caecomyces*, *Cyllamyces* and *Piromyce* were important AF the in rumen of cattle (Fig. 4). Previous studies have shown that the differences in AF of *Feramyces*, *Tahromyces*, *Buchfawromyces* and *Orpinomyces* did not have substrate preferences, and they can utilize a wider range of polysaccharides, including cellulose, xylose, glucose, starch, grass, and straw (32, 55 – 57). In addition, they may play important roles in the degradation of stubborn plant cell walls (55).

### Seasonal change in forage nutritional quality is a key driving factor affecting the rumen AF community

We used the ecological null model to identify the main driving factors affecting the dynamics of the rumen AF community of yak and cattle during different grazing months and seasons. We found that environmental selection and potential dispersal limitation were the main factors affecting the high turnover of rumen AF community in yak and cattle during different grazing seasons. The environmental selection was the dominant factor affecting the high turnover of rumen AF community in the warm season (Fig. 6). This may be the result of seasonal changes in the nutritional quality of forages since diet structure has a significant effect on the composition of AF communities (50, 51). Some studies have shown that fewer AF was observed in the rumen of ruminants fed in growing forage, while the number of AF increased in animals fed the mature forage of the same forage species (51, 58). It is noteworthy that around 35% of the seasonal changes in the rumen AF community in yak can be explained by climatic variables, forage nutrients, and rumen and host metabolisms, but these factors can only explain 21% of the seasonal variation in the cattle rumen AF (Fig. 5), suggesting that yak may be able to adjust the structure of rumen AF community according to the seasonal changes in climate and available nutrients for a better adaptation to the harsh conditions on the QTP.

To further reveal the environmental drivers of rumen AF community dissimilarities, we investigated whether climate variables, forage nutrition, rumen fermentation, and host metabolites contribute to rumen AF community dissimilarities in grazing yak and cattle. Based on the Mantel test and SEM, we found that forage physical-chemical properties including DM, NDF, and HC were key factors driving seasonal changes in the rumen AF community, especially the seasonal changes in DM of forage had the greatest contribution to the structure of rumen AF community (Fig. 6 and 7). Similarly, an earlier study found that the digestibility of DM in the rumen of sheep with fungi increased by 14 to 40% compared to the sheep without fungi (19). We further found that forage physical-chemical properties including DM, CF, ADL and Ash were positively correlated with *Cyllamyces*, *Feramyces*, *Tahromyces* and *Piromyces* (Fig. 5). Additionally, we observed a significant positive correlation between rumen fermentation and host metabolites and the relative abundances of cattle rumen AF, which may contribute to a better adaptation of cattle to the extreme environment on the QTP. Although SEM results can well explain the differences in the rumen AF community between grazing yak and cattle, it is not clear whether they can effectively explain the changes in other rumen microbial communities. In this study, we only collected the basic data on climate changes and forage nutrients in different grazing months. Other phenotypic data including feed intake, ligninolytic and cellulolytic enzyme activities, should be collected in future studies. Therefore, continuous future efforts should be dedicated to the genetic, physiological and metabolic characterization of the rumen AF community so that to further understand their function in the rumen microenvironment.

### Conclusions

Based on the ITS amplification sequencing and other environmental factors data, it is evident that grazing season, forage nutritional composition, and host animals species demonstrated important effects on the seasonal dynamics of rumen AF community. Particularly, we identified 12 AF genera, among which *Cyllamyces* and *Orpinomyces* were more abundant in the core rumen AF community of yak than cattle in different grazing months. We further found that the abundance and diversity of rumen AF were significantly higher in the cold season than warm season, while AF genera *Feramyces*, *Tahromyces,* and *Buwchfawromyces* were important seasonal indicators of rumen AF in grazing yak, and while *Caecomyces*, *Cyllamyces,* and *Piromyces* were of the grazing cattle. Factors such as forage DM, NDF, and HC were key factors driving seasonal dynamics in the rumen AF community of grazing yak and cattle. Therefore, this study provided novel evidence about seasonal changes in the rumen AF community of grazing livestock and the factors affecting ruminal AF under different environmental conditions. It also served as a scientific basis for further exploration of rumen microbial communities contributing to lignocellulose degradation in yak and cattle rumen.

## MATERIALS AND METHODS

### Experimental design and samples collection

The study area is located on the northeast edge of the QTP, which belongs to the transition zone between the QTP and the Loess Plateau, with an average elevation of around 3,000 m. This area has a typical alpine meadow pasture that is distributed with an average elevation of 3,300 m, annual average rainfall of 600 ~ 810 mm, annual average temperature of 4°C and rich water resources.

A total of 18 age-matched animals were used in this study, including nine 5-year-old female yak (Y) and nine 5-year-old local female cattle (C) from the Yangnuo Specialized Yak Breeding Cooperative (34°43'19.66"N, 102°28'49.51"E) at Xiahe county of Gannan Tibetan Autonomous Prefecture, Gansu Province, China. Animals were labeled and freed to browse in the same pasture without supplementary feeding for one year. Animals had free access to clean water throughout the experiment. Sample collection was performed from November 2018 to September 2019. The rumen fluid and blood samples of yak and cattle were collected during cold and warm seasons. Cold-season samples were collected during November (NovY and NovC), January (JanY and JanC), and March (MarY and MarC), while warm-season samples were collected during May (MayY and MayC), July (JulyY and JulyC) and September (SeptY and SeptC). Rumen fluid samples (*n* = 108, 50 mL/average per animal) were collected before the morning grazing by an oral stomach tube. The tube was thoroughly cleaned using fresh warm water between sample collections, and the initial 30 mL of the rumen fluid samples were discarded to avoid contamination with saliva. The rumen fluid samples were transferred in liquid nitrogen to the laboratory and stored at –80℃ for later analyses of VFAs and rumen AF diversity. The blood samples (*n* = 108, 10 mL from each animal) were collected from each animal by puncture of the jugular vein into non-oxalate tubes. Forage samples were also collected during the same time points for physical-chemical analysis. Long-term climate data of the study area was obtained from the Gansu Meteorological Station, including monthly mean air temperatures of daytime (DT) and night-time (NT), and monthly mean humidity (HD).

### Determination of physical-chemical properties of forage

Forage samples were collected from the pasture, in which animals grazed during the experimental stage. Ten quadrats (1 m^2^ square plot) with a distance greater than 10 m, were randomly placed in the pasture. To collect grass samples, forages were cut out of the ground part within the quadrats with scissors at six sample collection times. The forage samples from individual quadrats were oven-dried at 65°C to a constant weight, donated as the biomass of the forage, then ground to pass through a 1 mm screen, and stored at room temperature for further analysis. The physical-chemical properties of the forage samples, includingDM, CP, ether extract (EE), crude fiber (CF), cellulose, HC, nitrogen-free extract (NFE) and crude ash were determined using the procedures developed by the Association of Official Analytical Chemists (AOAC) (59). The contents of acid detergent fiber (ADF), acid detergent lignin (ADL), and neutral detergent fiber (NDF) were calculated according to Van Soest (60).

### Determination of rumen fermentation parameters and host blood metabolites

Total VFAs concentration was calculated as the sum of acetate (ACE), propionate (PRO), isobutyrate (IBUT), butyrate (BUT), iso valerate (IVAL) and valerate (VAL). For VFAs analysis, the cryopreserved rumen fluid samples were thawed at 4°C and thoroughly mixed by vertexing; centrifuged at 15,000 × *g* for 10 min at 4°C; and the supernatants were analyzed by the Agilent gas chromatography (7890A GC system Agilent Technologies Inc, Santa Clara, CA, USA) (61) with the chromatographic column of 100 m×0.250 mm×0.20 µm. Additionally, the blood was centrifuged at 1,500 × *g* (15 min at 4℃) (centrifuge, Eppendorf AG 22331 Hamburg; model of the rotor, Eppendorf F-35-6-30), and the supernatant (serum) was collected and transferred into new tubes for the subsequent biochemical analyses of the concentrations of serum GLU, triacylglycerols (TG), TC, lactate dehydrogenase (LDH), LDL-C, HDL-C and creatine kinase (CK) using the Mindray BS-240VET Automatic Hematology Analyzer (Mindray Corporation, Shenzhen, China). The activities of fatty acid synthase (FAS), hormone-sensitive lipase (HSL) and lipoprotein lipase (LPL) were analyzed using the enzyme-linked immunosorbent assay (ELISA) kits according to the instructions provided by the manufacturer (Shanghai Zhuocai, Shanghai, China). Serum NEFA concentrations were determined by a colorimetry assay kit (Nanjing Jiangcheng, Jiangsu, China) following the manufacturer’s instructions.

### DNA extraction, PCR amplification, and high-throughput sequencing

Total genomic DNA from rumen fluid samples (*n* = 108) was extracted using the Hexadecyl trimethyl Ammonium Bromide (CTAB) method (62). DNA concentration and purity were monitored on 1% agarose gel electrophoresis. The fungal ITS1 region was amplified by using primer pair ITS5-1737F (GGAAGTAAAAGTCGTAACAAGG)/ITS2-2043R (GCTGCGTTCTTCATCGATGC) (63). All PCR reactions were carried out in 30 µL reactions with 15 µL of the Phusion High-Fidelity PCR Master Mix (New England Biolabs), 0.2 µM of both forward and reverse primers, and around 10 ng template DNA. The thermal cycling condition consisted of an initial denaturation at 98℃ for 1 min, followed by 30 cycles of denaturation at 98℃ for 10 s, annealing at 50℃ for 30 s, and elongation at 72℃ for 30 s, and finished by extension at 72°C for 5 min. The PCR products were visualized in a 2% agarose gel and then purified with the GeneJET Gel Extraction Kit (Thermo Scientific). Sequencing libraries were generated using the Ion Plus Fragment Library Kit 48 rxns (Thermo Scientific) following the manufacturer’s recommendations. The library quality was assessed on the Qubit@ 2.0 Fluorometer (Thermo Scientific). Sequencing was performed on an Ion S5 XL platform (Thermo Scientific) at the Novogene Bioinformatics Technology Co., Ltd. (Tianjin, China), and 400 bp single-end reads were generated. Raw sequencing data have been deposited at the NCBI Sequence Read Archive (SRA) under BioProject ID PRJNA761685.

### ITS sequence analysis

Single-end reads were assigned to the samples based on their unique barcodes and truncated by cutting off the barcodes and primer sequences. Quality filtering was performed using Cutadapt (v1.9.1) (64). The quality-filtered reads were annotated with UNITE database (v8.2) (65) using the UCHIME algorithm (66) to detect chimera sequences resulting in 8,342,004 non-chimeric sequences. To obtain more operational taxonomic units (OTUs), the potential AF community in the rumen was explored for further analysis. Sequences with ≥97% similarity were assigned to the same OTUs using Uparse (v7.0.1001) (67), leading to 13,407 unique OTUs. Taxonomies were assigned to representative sequences using the blast algorithm implemented in QIIME (v1.9.1) (68). By comparing the 13,407 unique OTU sequences obtained from the UNITE database and the ITS sequences in the NCBI databases, we obtained 545 OTUs belonging to the phylum Neocallimastigomycota. Except for one yak sample collected in September faild in the annotation of the AF information, we annotated a total of 12 genera of the phylum Neocallimastigomycota in all other samples (*n* = 107).

### Diversity and correlation with the environmental factors

Alpha diversity such as Chao1 species richness and Shannon diversity index were estimated using QIIME and displayed with R software (v3.6.1). Alpha diversity of the rumen AF community in different grazing months and seasons was analyzed using non-parametric Friedman tests (69). For beta-diversity, the beta_diversity.py script in the QIIME pipeline was done using the Bray-Curtis distances matrices, and the principal coordinate analysis (PCoA) plot was plotted using the ggplot2 package (v3.1.1) (70). Permutational multivariate analysis of variance (PERMANOVA) was used to examine the differences in rumen AF community between yak and cattle during different grazing months. Moreover, intersections between sets of OTUs of rumen AF community in yak and cattle during different grazing months were visualized using the UpSet plot (with the R package UpSetR (v1.3.3) (71). The composition of the rumen AF community in different grazing months was visualized using Circos software (72). Detection of differentially abundant taxa between yak and cattle during different grazing months was done using Linear discriminant analysis (LDA) Effect Size (LEfSe). The LDA values was set at a default value of ≥2.0 and the *P*-value < 0.05 were considered significantly enriched (73).

Environmental metadata was z-score standardized and evaluated for collinearity using the Pearson correlation coefficient (74). Species relative abundances, climate factors, forage nutrition and metabolites of rumen microbiota and host blood were used for Bray-Curtis dbRDA using phyloseq package (75). The significance of each response variable was confirmed with an analysis of variance (ANOVA) in the dbRDA (anova.cca) function in the vegan R package (76). Only significant (*P*-value < 0.05) response variables were kept in the model. The explanatory value (in percentage) of significant response variables was assessed with a variation partitioning analysis of the vegan R package (76). For each environmental variable, including climate factors, forage nutrition and metabolites of rumen microbiota and host blood, a partial Mantel test was carried out to examine the correlation between environmental variable and the composition of rumen AF communities in yak and cattle during different grazing seasons (cold versus warm) using the vegan R package (76).

### Random forests model

Random forest machine learning was performed with a random forest package in R (77). Lists of taxa ranked by their importance were determined over 1,000 iterations. Samples were randomly selected at a ratio of 7:3 and divided into the training set and test set. The best combination of mtry (number of species variables) and ntree (number of decision trees) was identified by 10-fold cross-validation.

### Estimation of the stochasticity of community assembly

To assess the relative importance of the deterministic and stochastic processes driving rumen AF community assembly in yak and cattle during different grazing seasons, the null model analysis using abundance-based beta-diversity matrices was performed using the R code as described by Zhang et al. (78). For all cases where β-mean-nearest taxon distance (βMNTD) did not deviate significantly from the null model distribution, we calculated the Raup-Crick beta-diversity metric for each pair of communities after a total of 1,000 iterations (79) and the relative species abundance (RC_bray_) based on Stegen et al. (80). We compared observed RC_bray_ values with those of a random null model distribution to determine the importance of the different factors according to the following rules: RC_bray_ values between –0.95 and +0.95 indicated drift, RC_bray_ values > +0.95 indicated communities to be less similar than expected by chance as a result of dispersal limitation, and RC_bray_ values < –0.95 indicated communities to be more similar than expected by chance as a result of mass effects (79, 80).

### Structure equation modelings

Structure equation models (SEM) were used to show the relationship between environmental factors, forage nutrition, rumen fermentation and rumen AF community structure of yak and cattle. Alpha diversity measure included Chao1 and Shannon diversity indices, while beta diversity was represented by the PC1 from the Bray-Curtis distance-based principal coordinate analysis. We first considered a full model that included all reasonable pathways, and then, we sequentially eliminated non-significant pathways until we attained the final model in which all pathways were significant. Since there was no single universally accepted test of the overall goodness of fit for SEM, we used a χ^2^ test, the standardized root means square residual (SRMR), the CFI, the GFI and the AIC as criteria to test the goodness of the model fit (81). The model has a good fit when the χ^2^ is low, the CFI (>0.95), GFI (>0.90) are high and the SRMR is near 0 (SRMR values of ≤0.05 can be considered a good fit; values between 0.05 and 0.08 can be considered an adequate fit). The SEM-related analysis was performed using the Lavaan R package (82).

## Data Availability

Raw sequencing data have been deposited at the NCBI Sequence Read Archive (SRA) under BioProject ID PRJNA761685.

## AFTER EPUB

*[This article was published on 14 September 2023 with incorrect affiliations and a missing author contribution for Javad Gharechahi. The errors were corrected in the current version, posted on 21 September 2023.]*

## SUPPLEMENTAL MATERIAL

Fig. S1 spectrum.00788-23-s0001.tif Seasonal dynamics of environmental factors and forage nutrients in different grazing months.

Fig. S2 spectrum.00788-23-s0002.tif Composition of rumen fungal communities in yak and cattle during different grazing months.

Fig. S3 spectrum.00788-23-s0003.tif Effects of different grazing months on the diversity and structure of rumen anaerobic fungi community of yak and cattle.

Fig. S4 spectrum.00788-23-s0004.tif Composition of rumen anaerobic fungi community in yak and cattle during different grazing months.

Fig. S5 spectrum.00788-23-s0005.tif Effects of different grazing months on rumen anaerobic fungi in yak and cattle.

Fig. S6 spectrum.00788-23-s0006.tif The Pearson correlation between climatic variables, forage nutrients, and rumen and blood metabolites of yak collected in different grazing months.

Fig. S7 spectrum.00788-23-s0007.tif The Pearson correlation between climatic variables, forage nutrients, and rumen and blood metabolites of cattle collected in different grazing months.

Fig. S8 spectrum.00788-23-s0008.tif Variation partitioning analysis of the relative contributions of grazing months, seasons, and host animal species to the variation in rumen anaerobic fungi community.

Fig. S9 spectrum.00788-23-s0009.tif Beta−NTI distribution.

Tables S1 to S3 and legends to Fig. S1 to S9 spectrum.00788-23-s0010.docx Tables S1 (Rumen fermentation parameters of yak and cattle in different months and seasons), S2 (Adonis test for beta diversity of rumen anaerobic fungi community of yak and cattle in different grazing months), and S3 (Effects of climatic variables of different grazing months, forage nutrients, rumen and blood metabolites on the distribution of rumen anaerobic fungi in yak and cattle)

ASM does not own the copyrights to Supplemental Material that may be linked to, or accessed through, an article. The authors have granted ASM a non-exclusive, world-wide license to publish the Supplemental Material files. Please contact the corresponding author directly for reuse.
