# Supplementary figures and images for: Forage lignocellulose is an important factor in driving the seasonal dynamics of rumen anaerobic fungi in grazing yak and cattle
Source: Microbiol Spectr. 2023 Sep 14;11(5):e00788-23. doi: 10.1128/spectrum.00788-23 (PMC10581131; doi:10.1128/spectrum.00788-23)

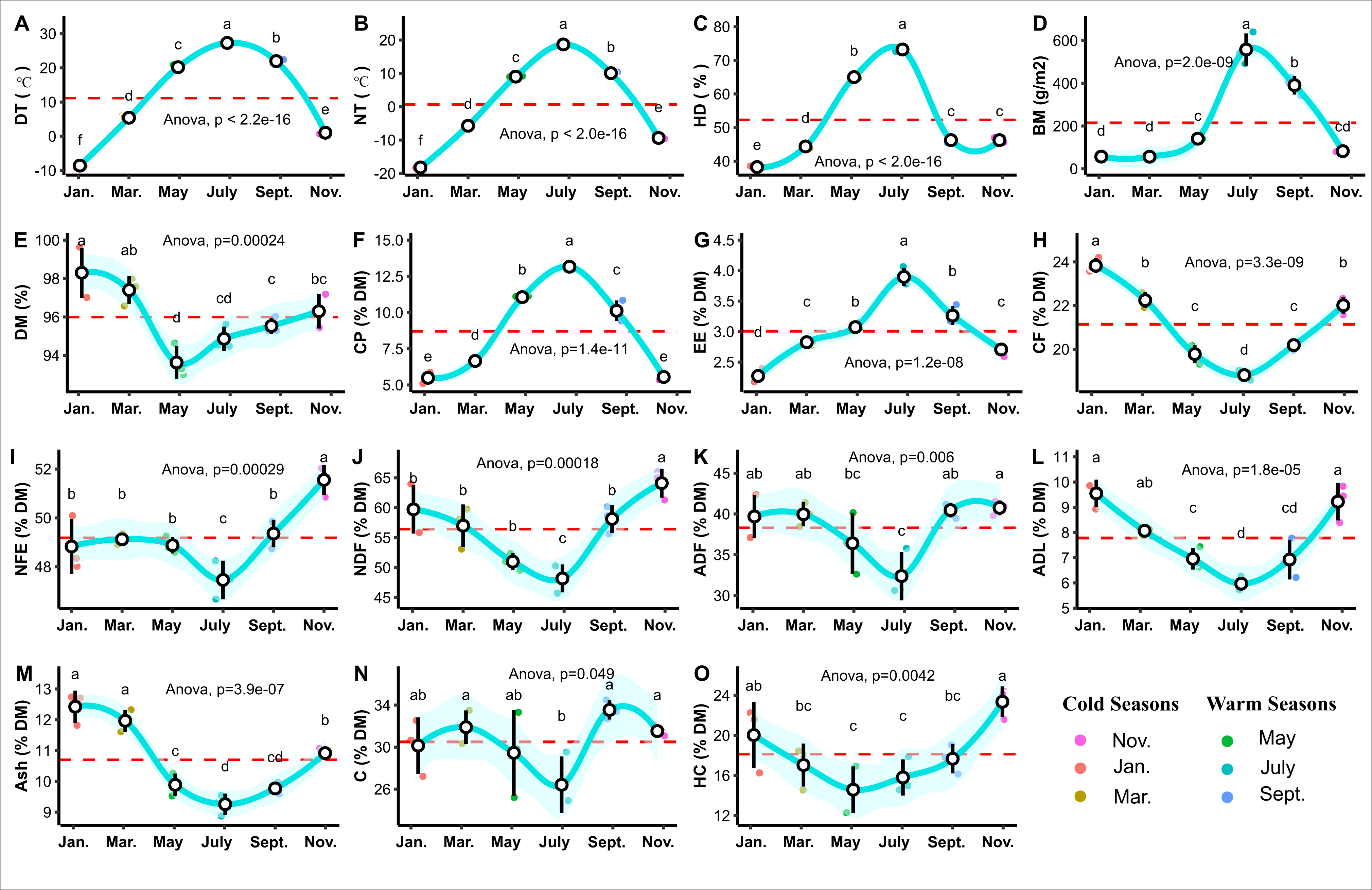

Supplement: Fig. S1 — Seasonal dynamics of environmental factors and forage nutrients in different grazing months. [file spectrum.00788-23-s0001.tif]

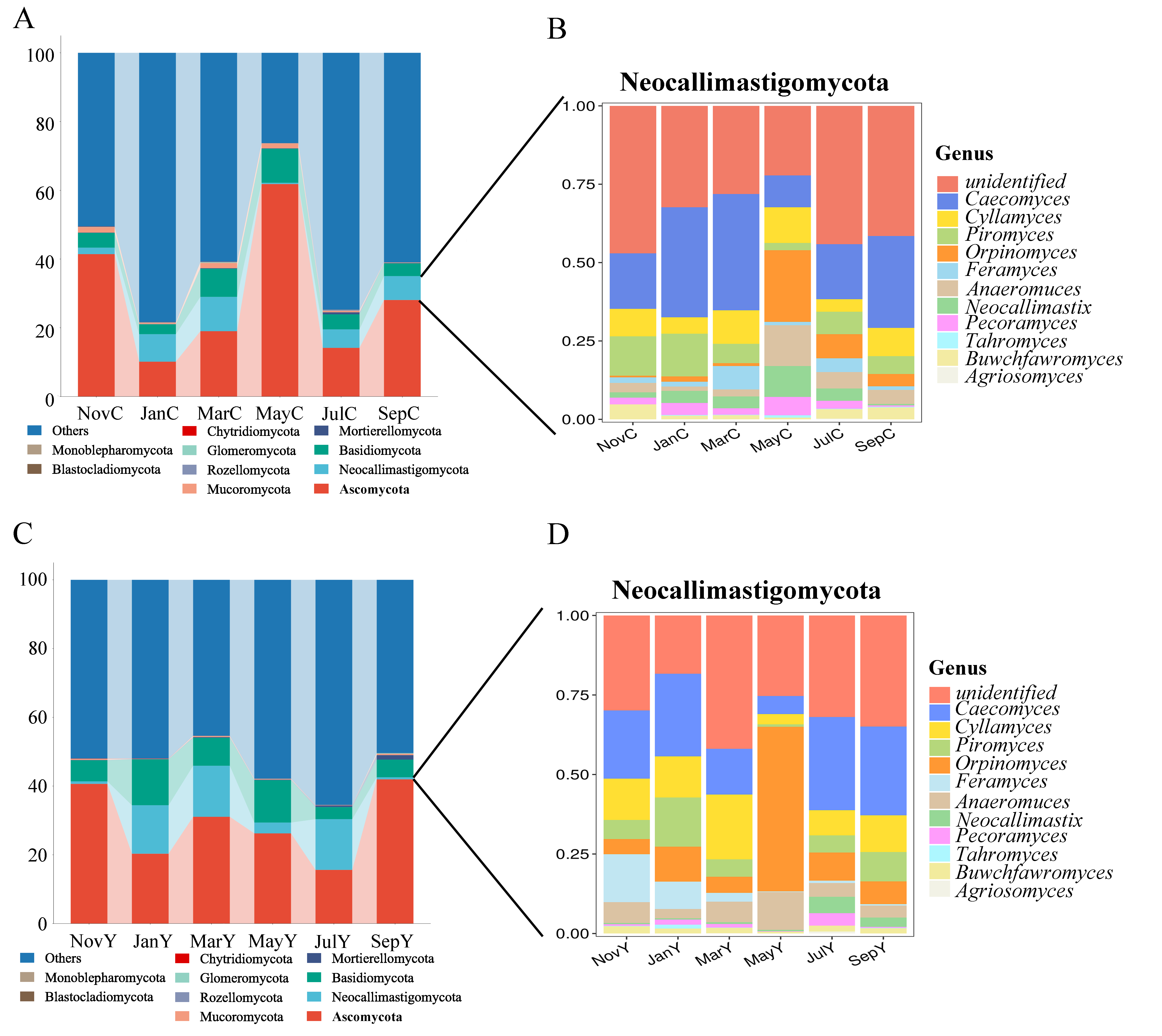

Supplement: Fig. S2 — Composition of rumen fungal communities in yak and cattle during different grazing months. [file spectrum.00788-23-s0002.tif]

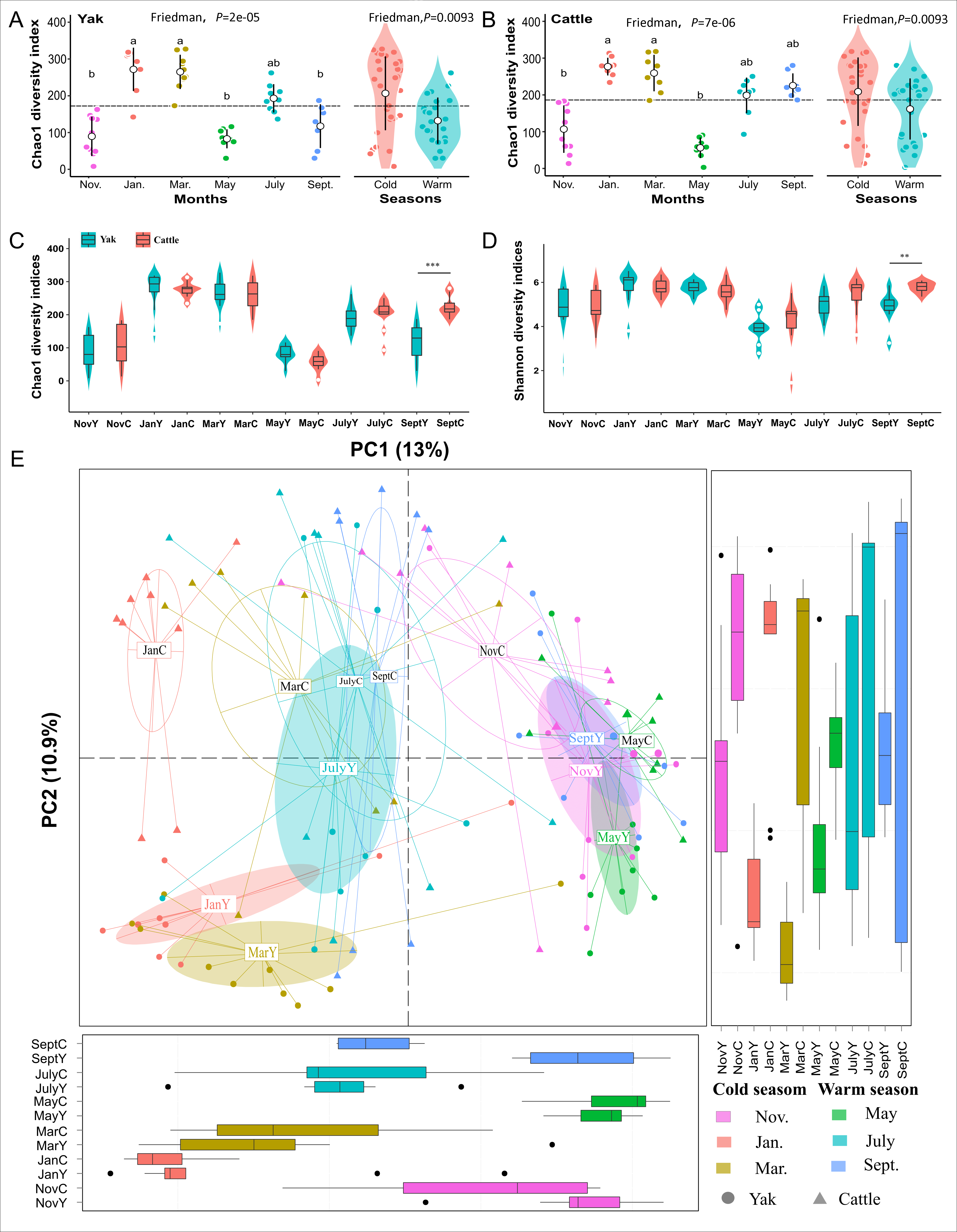

Supplement: Fig. S3 — Effects of different grazing months on the diversity and structure of rumen anaerobic fungi community of yak and cattle. [file spectrum.00788-23-s0003.tif]

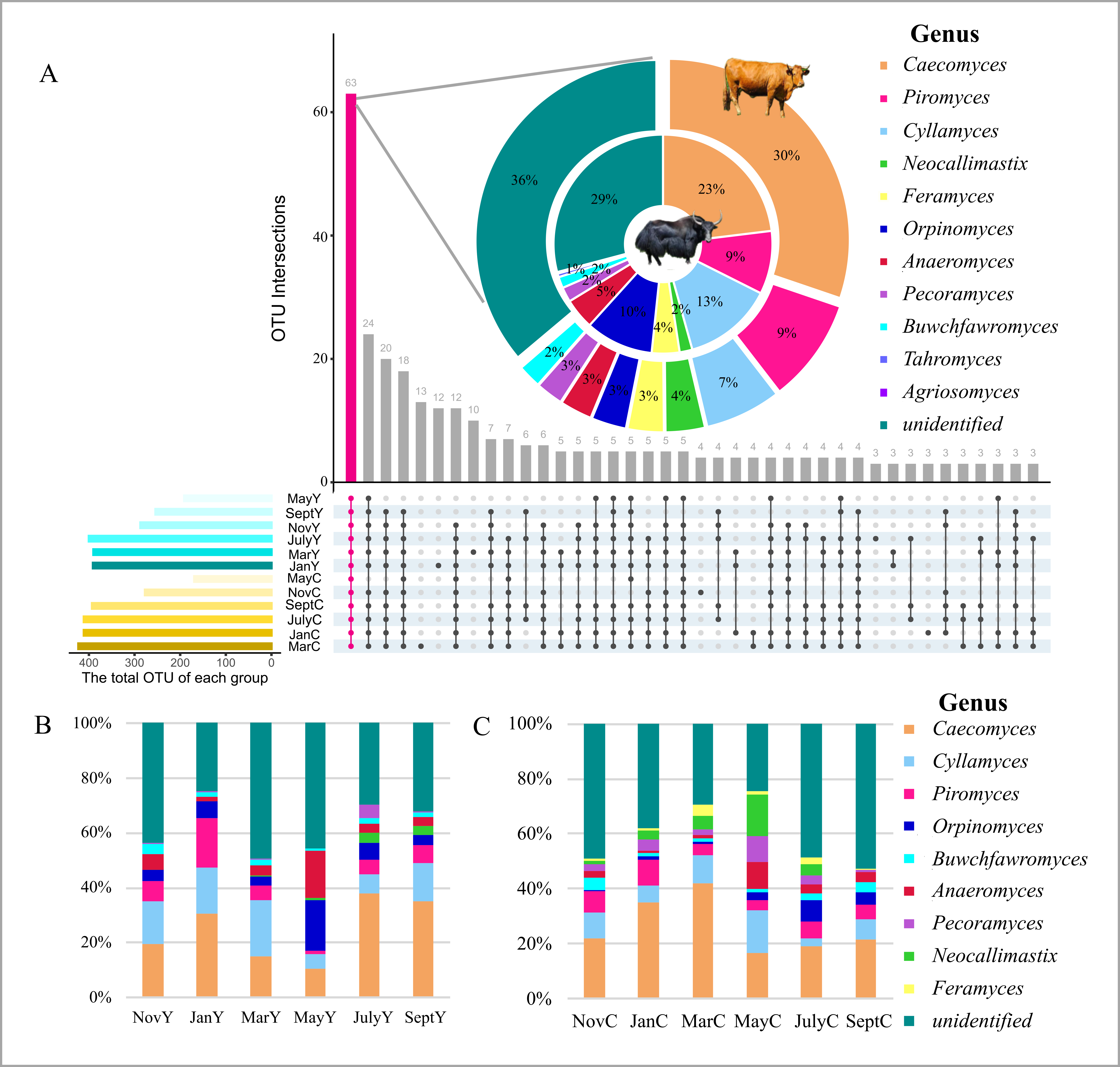

Supplement: Fig. S4 — Composition of rumen anaerobic fungi community in yak and cattle during different grazing months. [file spectrum.00788-23-s0004.tif]

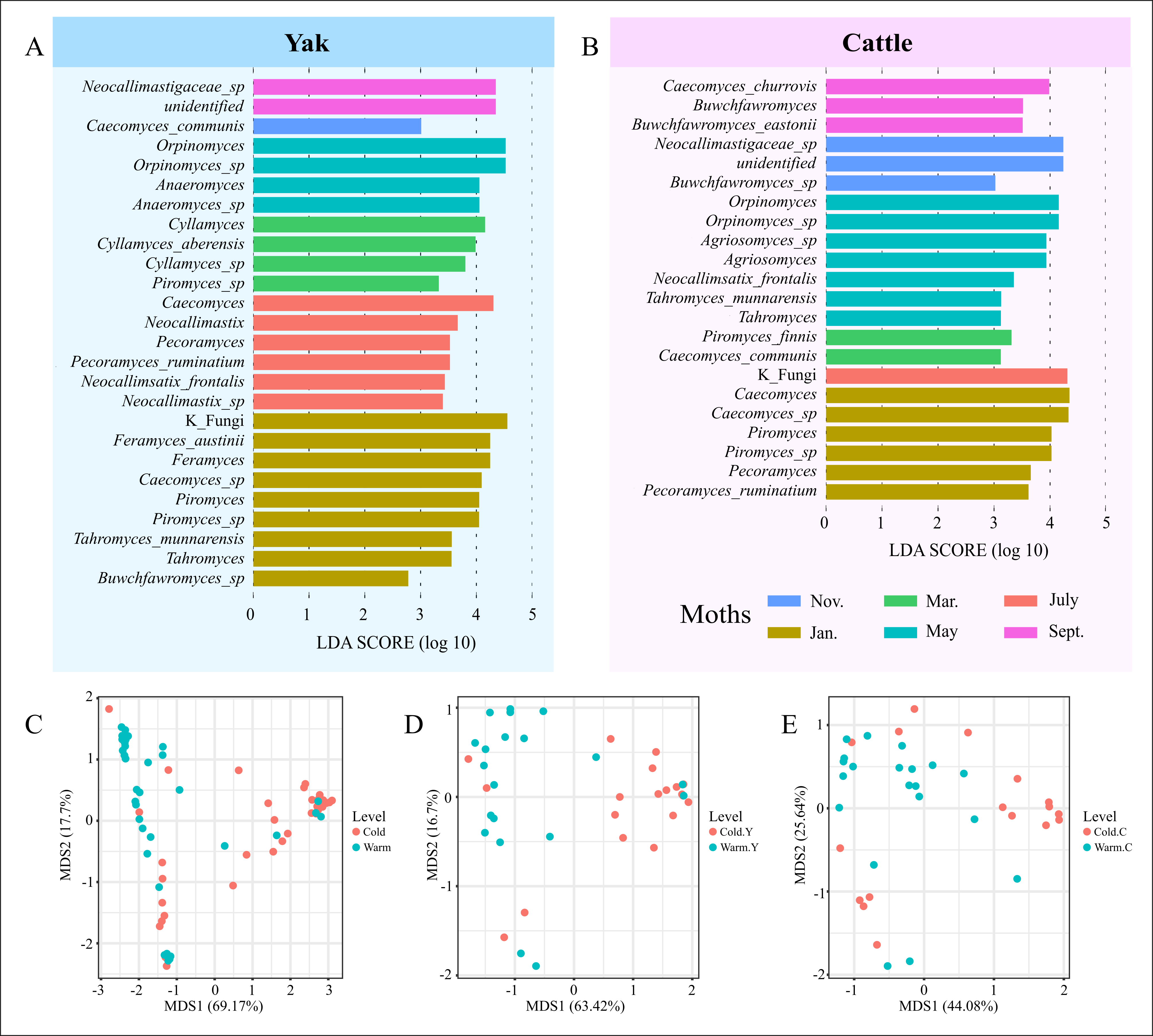

Supplement: Fig. S5 — Effects of different grazing months on rumen anaerobic fungi in yak and cattle. [file spectrum.00788-23-s0005.tif]

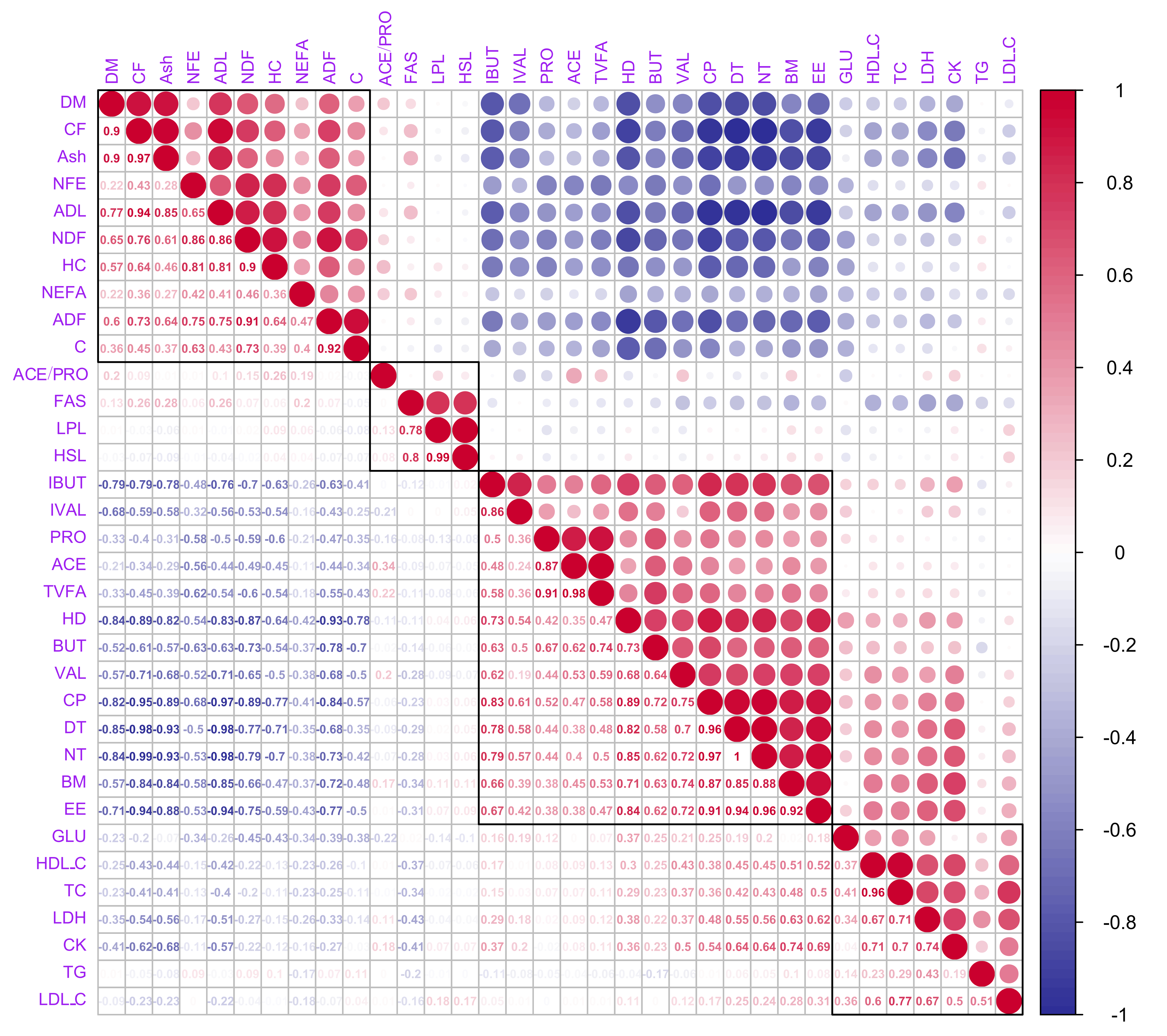

Supplement: Fig. S6 — The Pearson correlation between climatic variables, forage nutrients, and rumen and blood metabolites of yak collected in different grazing months. [file spectrum.00788-23-s0006.tif]

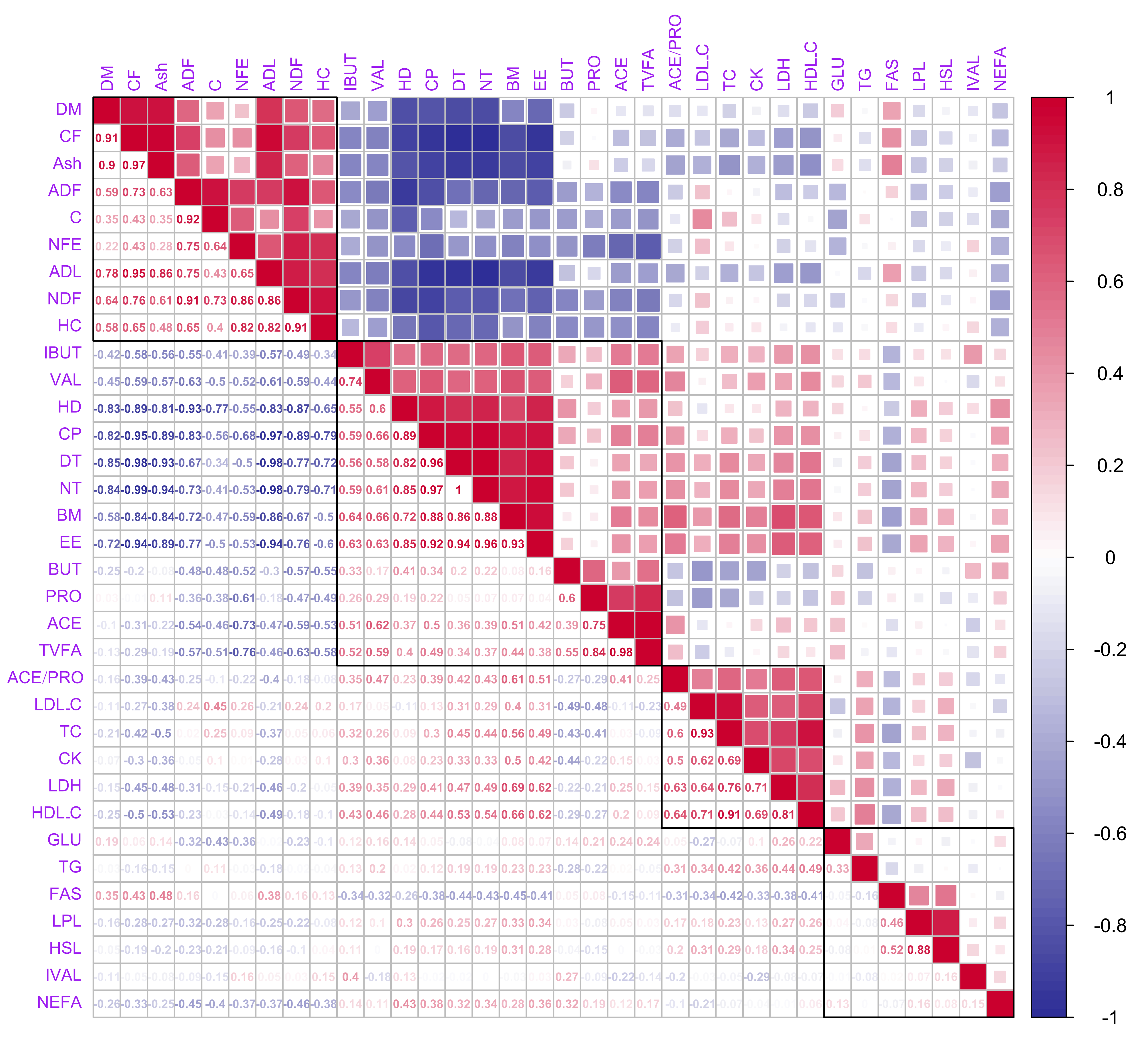

Supplement: Fig. S7 — The Pearson correlation between climatic variables, forage nutrients, and rumen and blood metabolites of cattle collected in different grazing months. [file spectrum.00788-23-s0007.tif]

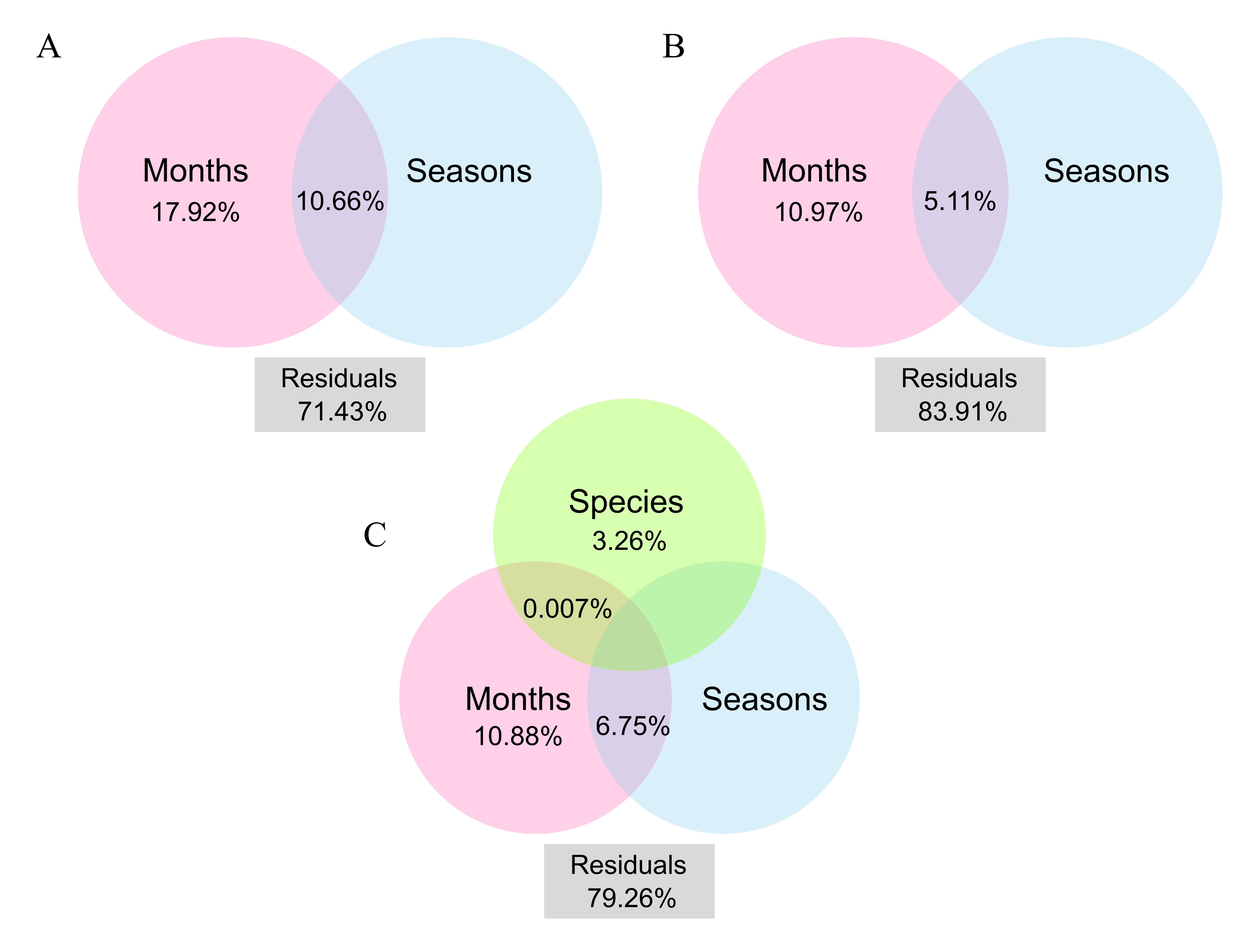

Supplement: Fig. S8 — Variation partitioning analysis of the relative contributions of grazing months, seasons, and host animal species to the variation in rumen anaerobic fungi community. [file spectrum.00788-23-s0008.tif]

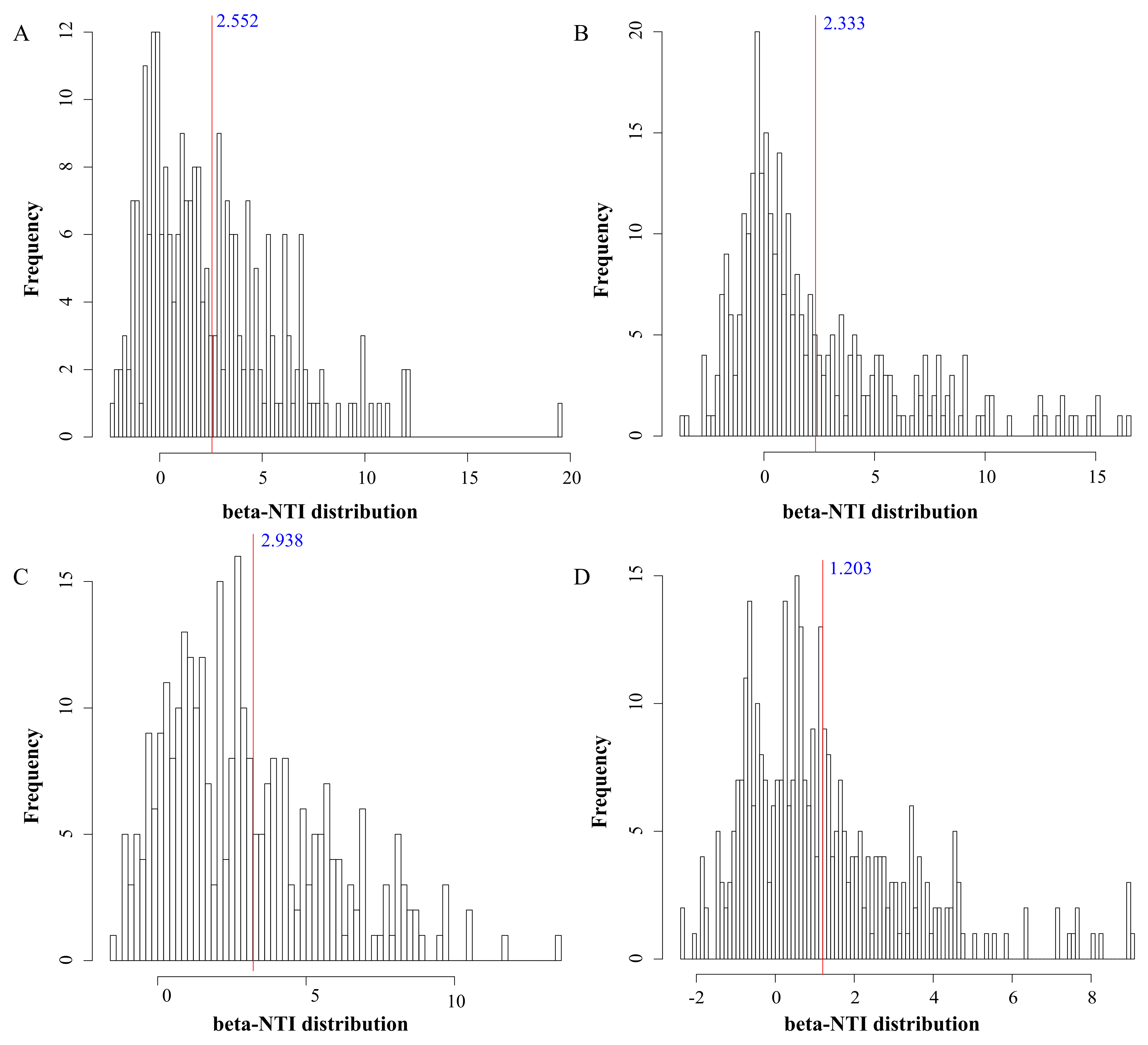

Supplement: Fig. S9 — Beta−NTI distribution. [file spectrum.00788-23-s0009.tif]
