# Supplementary material for: Forage lignocellulose is an important factor in driving the seasonal dynamics of rumen anaerobic fungi in grazing yak and cattle
Source: Microbiol Spectr. 2023 Sep 14;11(5):e00788-23. doi: 10.1128/spectrum.00788-23 (PMC10581131; doi:10.1128/spectrum.00788-23)
Supplement: Tables S1 to S3 and legends to Fig. S1 to S9 — Tables S1 (Rumen fermentation parameters of yak and cattle in different months and seasons), S2 (Adonis test for beta diversity of rumen anaerobic fungi community of yak and cattle in different grazing months), and S3 (Effects of climatic variables of different grazing months, forage nutrients, rumen and blood metabolites on the distribution of rumen anaerobic fungi in yak and cattle) [file spectrum.00788-23-s0010.docx]

**Forage** **lignocellulose is an important factor in driving the seasonal dynamics of** **rumen anaerobic** **fungi in grazing yak and cattle**

Zeyi Liang^a, b †^, Anum Ali Ahmad^a, b †^, Jianbo Zhang^a^^, b†^, Mei Du^a^, Juanshan Zheng^a^, Peng Wang^b^, Jianlin Han^c, d^, Ping Yan^a^^[[1]](#footnote-1)^*, Ghasem Hosseini Salekdeh^e, f*^, Xuezhi Ding^a, b^*

1. Key Laboratory of Yak Breeding Engineering, Lanzhou Institute of Husbandry and Pharmaceutical Sciences, Chinese Academy of Agricultural Sciences, Lanzhou 730050, China.
2. Key Laboratory of Veterinary Pharmaceutical Development, Ministry of Agricultural and Rural Affairs, Lanzhou Institute of Husbandry and Pharmaceutical Sciences, Chinese Academy of Agricultural Sciences, Lanzhou 730050, China.
3. Livestock Genetics Program, International Livestock Research Institute (ILRI), Nairobi 00100, Kenya.
4. CAAS-ILRI Joint Laboratory on Livestock and Forage Genetic Resources, Institute of Animal Science, Chinese Academy of Agricultural Sciences (CAAS), Beijing 100193, China.
5. Department of Systems Biology, Agricultural Biotechnology Research Institute of Iran, Agricultural Research, Education, and Extension Organization, Karaj, Iran.
6. Department of Molecular Sciences, Macquarie University, North Ryde, NSW, Australia.

**Running Title:** Seasonal dynamics of rumen anaerobic fungi in yak

Supplement Material

Tables

**Table S1** Rumen fermentation parameters of yak and cattle in different months and seasons

| **Items** | **Breed** | **Cold season** | | | |  | **Warm season** | | | | **AVG** | **SEM** | ***P*-value** | | |
| --- | --- | --- | --- | --- | --- | --- | --- | --- | --- | --- | --- | --- | --- | --- | --- |
|  |  | **Nov.** | **Jan.** | **Mar.** | **avg** |  | **May** | **July** | **Sept.** | **avg** |  |  | **Species** | **Seasons** | **Months** |
| Rumen fermentation parameters | | | | | | | | | | | | | | | |
| ACE, mmol/L | C | 37.14^c^ | 49.59^b^ | 46.52^b^ | 44.07^B^ |  | 46.15^b^ | 56.02^a^ | 47.82^b^ | 50.23^A^ | 47.03 | 1.09 | 0.078 | 0.004 | <0.001 |
|  | Y | 41.24^c^ | 50.98^ab^ | 47.53^bc^ | 46.43^B^ |  | 51.09^ab^ | 57.36^a^ | 51.99^ab^ | 53.48^A^ | 49.83 | 1.12 |  | 0.001 | <0.001 |
| PRO, mmol/L | C | 7.80^c^ | 10.86^a^ | 10.19^a^ | 9.53 |  | 10.50^a^ | 10.40^a^ | 8.94^b^ | 9.96 | 9.74 | 0.22 | 0.25 | 0.32 | <0.001 |
|  | Y | 8.22^b^ | 9.97^a^ | 10.00^a^ | 9.38^B^ |  | 11.17^a^ | 11.31^a^ | 10.14^a^ | 10.87^A^ | 10.10 | 0.22 |  | <0.001 | <0.001 |
| BUT, mmol/L | C | 4.09^c^ | 5.49^b^ | 5.58^b^ | 5.00 |  | 6.66^a^ | 5.94^ab^ | 4.22^c^ | 5.62 | 5.23 | 0.18 | 0.92 | 0.085 | <0.001 |
|  | Y | 4.03^c^ | 5.01^c^ | 4.14^c^ | 4.37^B^ |  | 6.34^b^ | 7.84^a^ | 4.88^c^ | 6.35^A^ | 5.33 | 0.22 |  | <0.001 | <0.001 |
| IBUT, mmol/L | C | 0.47^bc^ | 0.45^c^ | 0.46^bc^ | 0.46^B^ |  | 0.51^bc^ | 0.66^a^ | 0.55^bc^ | 0.58^A^ | 0.52^B^ | 0.02 | <0.001 | <0.001 | <0.001 |
|  | Y | 0.46^b^ | 0.47^b^ | 0.43^b^ | 0.45^B^ |  | 0.98^a^ | 0.92^a^ | 0.86^a^ | 0.92^A^ | 0.68^A^ | 0.04 |  | <0.001 | <0.001 |
| VAL, mmol/L | C | 0.29^c^ | 0.33^bc^ | 0.29^c^ | 0.30^B^ |  | 0.44^b^ | 0.65^a^ | 0.44^b^ | 0.52^A^ | 0.41 | 0.03 | 0.79 | <0.001 | <0.001 |
|  | Y | 0.24^c^ | 0.24^c^ | 0.24^c^ | 0.24^B^ |  | 0.50^b^ | 0.78^a^ | 0.56^b^ | 0.61^A^ | 0.42 | 0.04 |  | <0.001 | <0.001 |
| IVAL, mmol/L | C | 0.55 | 0.43 | 0.43 | 0.47 |  | 0.48 | 0.49 | 0.39 | 0.46 | 0.46^B^ | 0.02 | <0.001 | 0.69 | 0.21 |
|  | Y | 0.44^c^ | 0.47^c^ | 0.42^c^ | 0.44^B^ |  | 1.19^a^ | 0.83^b^ | 0.81^b^ | 0.94^A^ | 0.68^A^ | 0.05 |  | <0.001 | <0.001 |
| TVFA, mmol/L | C | 50.35^c^ | 67.15^b^ | 63.48^b^ | 59.84^B^ |  | 64.74^b^ | 74.13^a^ | 62.36^b^ | 67.36^A^ | 63.45 | 1.37 | 0.086 | 0.0048 | <0.001 |
|  | Y | 54.61^c^ | 67.14^b^ | 62.75^bc^ | 61.31^B^ |  | 71.26^ab^ | 79.04^a^ | 69.23^b^ | 73.18^A^ | 67.03 | 1.53 |  | <0.001 | <0.001 |
| ACE/PRO, % | C | 4.76^b^ | 4.62^b^ | 4.59^b^ | 4.67^B^ |  | 4.40^b^ | 5.37^a^ | 5.35^a^ | 5.06^A^ | 4.85 | 0.07 | 0.31 | 0.007 | <0.001 |
|  | Y | 5.01^a^ | 5.12^a^ | 4.75^ab^ | 4.96 |  | 4.59^b^ | 5.09^a^ | 5.13^a^ | 4.94 | 4.95 | 0.05 |  | 0.85 | 0.01 |
| Carbohydrate metabolism Index | | | | | | | | | | | | | | | |
| GLU, mmol/L | C | 2.63^bc^ | 3.15^a^ | 2.99^ab^ | 2.91 |  | 2.63^bc^ | 3.30^a^ | 2.37^c^ | 2.79 | 2.85^A^ | 0.08 | 0.012 | 0.44 | 0.0018 |
|  | Y | 2.08^c^ | 2.3^bc^ | 2.89^ab^ | 2.43 |  | 3.14^a^ | 2.88^ab^ | 1.73^c^ | 2.59 | 2.50^B^ | 0.11 |  | 0.46 | <0.001 |
| LDH, U/L | C | 904.25^b^ | 706.32^cd^ | 848.41^bc^ | 821.70^B^ |  | 583.61^d^ | 1268.61^a^ | 1143.71^a^ | 1009.40^A^ | 912.00 | 39.15 | 0.0048 | 0.015 | <0.001 |
|  | Y | 750.35^b^ | 508.68^c^ | 664.77^bc^ | 645.84^B^ |  | 644.03^bc^ | 1029.21^a^ | 982.35^a^ | 885.20^A^ | 761.20 | 34.9 |  | <0.001 | <0.001 |
| CK, U/L | C | 216.56^bc^ | 138.58^de^ | 175.54^cd^ | 178.41 |  | 99.72^e^ | 257.73^ab^ | 290.55^a^ | 217.67 | 197.29 | 12.28 | 0.17 | 0.11 | <0.001 |
|  | Y | 175.48^b^ | 75.61^d^ | 124.67^c^ | 126.96^B^ |  | 124.51^c^ | 259.46^a^ | 288.06^a^ | 224.01^A^ | 173.75 | 11.61 |  | <0.001 | <0.001 |
| Lipid metabolism Index | | | | | | | | | | | | | | | |
| LDL-C, mmol/L | C | 0.89^b^ | 0.37^d^ | 0.58^c^ | 0.62 |  | 0.33^d^ | 0.73^bc^ | 1.42^a^ | 0.82 | 0.72^A^ | 0.06 | <0.001 | 0.078 | <0.001 |
|  | Y | 0.44 | 0.28 | 0.44 | 0.39 |  | 0.32 | 0.5 | 0.48 | 0.43 | 0.41^B^ | 0.03 |  | 0.40 | 0.07 |
| HDL-C, mmol/L | C | 1.76^b^ | 1.27^c^ | 1.66^b^ | 1.57^B^ |  | 1.31^c^ | 2.3^a^ | 2.33^a^ | 1.99^A^ | 1.77 | 0.07 | 0.34 | 0.0028 | <0.001 |
|  | Y | 1.63^bc^ | 1.14^c^ | 1.57^bc^ | 1.46^B^ |  | 1.38^c^ | 2.21^a^ | 2.07^ab^ | 1.88^A^ | 1.66 | 0.08 |  | 0.0087 | <0.001 |
| TC, mmol/L | C | 3.14^b^ | 1.78^d^ | 2.46^c^ | 2.48^B^ |  | 1.82^d^ | 3.42^b^ | 4.57^a^ | 3.28^A^ | 2.87^A^ | 0.15 | <0.001 | 0.0087 | <0.001 |
|  | Y | 2.16^bc^ | 1.48^c^ | 2.1^bc^ | 1.93^B^ |  | 1.77^c^ | 2.87^a^ | 2.6^ab^ | 2.42^A^ | 2.16^B^ | 0.11 |  | 0.023 | <0.001 |
| NEFA, µmol/L | C | 483.18^b^ | 498.01^b^ | 548.72^ab^ | 509.44 |  | 578.84^ab^ | 633.96^a^ | 488.41^b^ | 569.75 | 538.43^A^ | 15.98 | 0.0068 | 0.059 | 0.023 |
|  | Y | 531.29^a^ | 515.39^a^ | 463.59^a^ | 504.93 |  | 464.74^a^ | 356.88^b^ | 519.04^a^ | 447.70 | 477.33^B^ | 15.33 |  | 0.061 | 0.0058 |
| FAS, nmol/L | C | 3.14ab | 4.43a | 4.25a | 3.90^A^ |  | 3.38ab | 2.20b | 1.07b | 2.24^B^ | 3.10 | 0.27 | 0.92 | 0.0016 | 0.005 |
|  | Y | 3.25ab | 4.22a | 3.47a | 3.63 |  | 4.01a | 2.06ab | 1.18b | 2.44 | 3.06 | 0.31 |  | 0.055 | 0.036 |
| TG, mmol/L | C | 0.26 | 0.22 | 0.28 | 0.25 |  | 0.22 | 0.28 | 0.31 | 0.27 | 0.26 | 0.01 | 0.6 | 0.45 | 0.13 |
|  | Y | 0.29 | 0.24 | 0.26 | 0.26 |  | 0.22 | 0.27 | 0.34 | 0.28 | 0.27 | 0.02 |  | 0.62 | 0.41 |
| LPL, U/L | C | 238.95 | 195.32 | 212.31 | 216.45 |  | 204.99 | 332.28 | 220.69 | 257.17 | 236.03 | 15.79 | 0.25 | 0.20 | 0.12 |
|  | Y | 234.39 | 215.74 | 187.05 | 212.46 |  | 181.22 | 241.41 | 199.23 | 209.25 | 210.92 | 15.19 |  | 0.92 | 0.79 |
| HSL, ng/mL | C | 3.10 | 2.55 | 2.56 | 2.75 |  | 2.09 | 3.95 | 2.87 | 3.01 | 2.88 | 0.19 | 0.47 | 0.50 | 0.084 |
|  | Y | 2.88 | 2.65 | 2.41 | 2.65 |  | 2.48 | 3.03 | 2.57 | 2.71 | 2.68 | 0.20 |  | 0.87 | 0.93 |

^a, b, c, d, e,^ Different small letter superscripts represent significant difference between different grazing months for yak and cattle, respectively (P < 0.05). ^A, B^ Different capital letter superscripts represent significant difference between species and seasons, respectively (P < 0.05). AVG: average. SEM: standard error of the mean. ACE, acetic acid; PRO, propionic acid; BUT, butyric acid; IBUT, isobutyric acid; VAL, valeric acid; IVAL, isovaleric acid; TVAF, total volatile fatty acids; ACE/PRO, acetic acid / propionic acid; GLU, glucose; LDH, lactate dehydrogenase; CK, Creatine Kinase; LDL-C, low density lipoprotein cholesterol; HDL-C, High density lipoprotein cholesterol; TC, total cholesterol; NEFA, non-esterified fatty acids; FAS, fatty acid synthase; TG, triglyceride; LPL, lipoprotein lipase; HSL, hormone-sensitive lipase.

**Table S2** Adonis test for beta diversity of rumen AF community of yak and cattle in different grazing months

| **Sample Type** | **Vs. Group** | **Df** | **SumsOfSqs** | **MeanSqs** | **F.Model** | **R^2^** | **Pr(>F)** |
| --- | --- | --- | --- | --- | --- | --- | --- |
| Yak | JanY vs. MarY | 1(17) | 0.6996(3.4085) | 0.6996(0.2005) | 3.489 | 0.1703(0.8297) | 0.001 |
|  | JanY vs. MayY | 1(16) | 1.9644(4.3820) | 1.9644(0.2739) | 7.173 | 0.3095(0.6905) | 0.001 |
|  | JanY vs. JulyY | 1(16) | 1.0413(4.3076) | 1.0413(0.2692) | 3.868 | 0.1947(0.8053) | 0.001 |
|  | JanY vs. SeptY | 1(16) | 1.6765(5.0445) | 1.6765(0.3153) | 5.318 | 0.2494(0.7506) | 0.001 |
|  | JanY vs. NovY | 1(17) | 1.7031(5.0782) | 1.7031(0.2987) | 5.701 | 0.2511(0.7489) | 0.001 |
|  | MarY vs. MayY | 1(17) | 1.8383(4.5743) | 1.8383(0.2691) | 6.832 | 0.2867(0.7133) | 0.001 |
|  | MarY vs. JulyY | 1(17) | 1.1032(4.4999) | 1.1033(0.2647) | 4.168 | 0.1969(0.8031) | 0.001 |
|  | MarY vs. SeptY | 1(17) | 1.7035(5.2368) | 1.7035(0.3081) | 5.530 | 0.2455(0.7545) | 0.001 |
|  | MarY vs. NovY | 1(18) | 1.6947(5.2705) | 1.6947(0.2928) | 5.788 | 0.2433(0.7567) | 0.001 |
|  | MayY vs. JulyY | 1(16) | 1.2815(5.4735) | 1.2815(0.3421) | 3.746 | 0.1897(0.8103) | 0.001 |
|  | MayY vs. SeptY | 1(16) | 0.7369(6.2103) | 0.7369(0.3882) | 1.899 | 0.1061(0.8939) | 0.007 |
|  | MayY vs. NovY | 1(17) | 0.9607(6.2441) | 0.9608(0.3673) | 2.616 | 0.1334(0.8667) | 0.003 |
|  | JulyY vs. SeptY | 1(16) | 1.0541(6.1359) | 1.0541(0.3835) | 2.749 | 0.1466(0.8534) | 0.001 |
|  | JulyY vs. NovY | 1(17) | 1.2428(6.1697) | 1.2428(0.3629) | 3.424 | 0.1677(0.8323) | 0.001 |
|  | SeptY vs. NovY | 1(17) | 0.4950(6.9065) | 0.4950(0.4063) | 1.219 | 0.0669(0.9331) | 0.169 |
| Cattle | JanC vs. MarC | 1(15) | 0.6979(3.3668) | 0.6979(0.2245) | 3.109 | 0.1717(0.8283) | 0.001 |
|  | JanC vs. MayC | 1(15) | 2.0045(4.1250) | 2.0045(0.2750) | 7.289 | 0.3270(0.6730) | 0.001 |
|  | JanC vs. JulyC | 1(16) | 0.8516(4.1942) | 0.8516(0.2621) | 3.249 | 0.1688(0.8312) | 0.001 |
|  | JanC vs. SeptC | 1(15) | 1.2363(3.6628) | 1.2363(0.2442) | 5.063 | 0.2524(0.7477) | 0.001 |
|  | JanC vs. NovC | 1(17) | 1.5342(4.8498) | 1.5342(0.2853) | 5.378 | 0.2403(0.7597) | 0.001 |
|  | MarC vs. MayC | 1(14) | 1.3919(4.8290) | 1.3919(0.3449) | 4.035 | 0.2237(0.7763) | 0.001 |
|  | MarC vs. JulyC | 1(15) | 0.5476(4.8981) | 0.5476(0.3265) | 1.677 | 0.1006(0.8995) | 0.075 |
|  | MarC vs. SeptC | 1(14) | 0.7215(4.3668) | 0.7215(0.3119) | 2.313 | 0.1418(0.8582) | 0.011 |
|  | MarC vs. NovC | 1(16) | 0.8962(5.5537) | 0.8962(0.3471) | 2.582 | 0.1390(0.8611) | 0.003 |
|  | MayC vs. JulyC | 1(15) | 1.1620(5.6564) | 1.1620(0.3771) | 3.081 | 0.1704(0.8296) | 0.002 |
|  | MayC vs. SeptC | 1(14) | 1.2668(5.1250) | 1.2668(0.3661) | 3.461 | 0.1982(0.8018) | 0.001 |
|  | MayC vs. NovC | 1(16) | 0.8815(6.3119) | 0.8815(0.3945) | 2.234 | 0.1225(0.8775) | 0.004 |
|  | JulyC vs. SeptC | 1(15) | 0.5154(5.1941) | 0.5154(0.3463) | 1.488 | 0.0903(0.9097) | 0.113 |
|  | JulyC vs. NovC | 1(17) | 0.7427(6.3811) | 0.7427(0.3754) | 1.979 | 0.1043(0.8957) | 0.009 |
|  | SeptC vs. NovC | 1(16) | 0.6812(5.8497) | 0.6812(0.3656) | 1.863 | 0.1043(0.8957) | 0.03 |
| Yak vs. Cattle | JanY vs. JanC | 1(16) | 1.0470(2.9395) | 1.0470(0.1837) | 5.699 | 0.2626(0.7374) | 0.001 |
|  | MarY vs. MarC | 1(16) | 1.1379(3.8358) | 1.1380(0.2397) | 4.747 | 0.2288(0.7712) | 0.001 |
|  | MayY vs. MayC | 1(15) | 0.7941(5.5675) | 0.7941(0.3712) | 2.140 | 0.1248(0.8752) | 0.01 |
|  | JulyY vs. JulyC | 1(16) | 0.4537(5.5623) | 0.4537(0.3476) | 1.305 | 0.0754(0.9246) | 0.167 |
|  | SeptY vs. SeptC | 1(15) | 0.8946(5.7678) | 0.8947(0.3845) | 2.327 | 0.1343(0.8657) | 0.004 |
|  | NovY vs. NovC | 1(18) | 0.7127(6.9885) | 0.7127(0.3883) | 1.836 | 0.0926(0.9075) | 0.016 |

Df: degrees of freedom; SumsOfSqs: Total variance, also known as the sum of squares of deviations; Sqs: Mean Square (variance), SumsOfSqs/DF; F. Model: F test value; R^2^ indicates the degree of interpretation of the sample difference for different groups, i. e. the ratio of the group variance to the total variance. The larger R^2^ is, the higher the degree of interpretation of the sample difference for different groups; PR is P value, less than 0.05 indicates the reliability of this test. Inside the parentheses are the values corresponding to the residuals.

**Table S3** Effects of climatic variables of different grazing months, forage nutrients, rumen microbial metabolites and host blood metabolites on the distribution of rumen anaerobic fungi in yak and cattle

| **Group** | **Factors** | **Yak** | | | |  | **Cattle** | | | |
| --- | --- | --- | --- | --- | --- | --- | --- | --- | --- | --- |
|  |  | **RDA1** | **RDA2** | **R^2^** | **P****-value** |  | **RDA1** | **RDA2** | **R^2^** | **P-value** |
| **Environment variables** | DT | -0.9020 | 0.4318 | 0.2135 | 0.0045 |  | -0.7957 | -0.6057 | 0.2576 | 0.0005 |
|  | NT | -0.8973 | 0.4414 | 0.1816 | 0.0070 |  | -0.7828 | -0.6223 | 0.2522 | 0.0005 |
|  | HD | -0.9983 | -0.0585 | 0.1300 | 0.0365 |  | -0.8324 | -0.5542 | 0.3755 | 0.0005 |
| **Forage nutrients** | BM | -0.1343 | 0.9909 | 0.0534 | 0.2494 |  | -0.3248 | -0.9458 | 0.0977 | 0.0730 |
|  | DM | 0.9995 | 0.0313 | 0.3545 | 0.0005 |  | 0.8333 | 0.5528 | 0.5739 | 0.0005 |
|  | CP | -0.9765 | 0.2157 | 0.1549 | 0.0145 |  | -0.7890 | -0.6144 | 0.2510 | 0.0010 |
|  | EE | -0.5821 | 0.8132 | 0.1025 | 0.0700 |  | -0.7129 | -0.7012 | 0.1554 | 0.0155 |
|  | CF | 0.9399 | -0.3414 | 0.1884 | 0.0070 |  | 0.7846 | 0.6200 | 0.3387 | 0.0005 |
|  | NFE | 0.9946 | 0.1043 | 0.0144 | 0.7036 |  | 0.9947 | -0.1031 | 0.0067 | 0.8376 |
|  | NDF | 0.9984 | 0.0571 | 0.1469 | 0.0180 |  | 0.9186 | 0.3952 | 0.1967 | 0.0055 |
|  | ADF | 0.9523 | 0.3052 | 0.0355 | 0.3958 |  | 0.8128 | 0.5826 | 0.1704 | 0.0090 |
|  | ADL | 0.8971 | -0.4419 | 0.1887 | 0.0080 |  | 0.8333 | 0.5529 | 0.1971 | 0.0035 |
|  | Ash | 0.9405 | -0.3398 | 0.1428 | 0.0210 |  | 0.7008 | 0.7134 | 0.3507 | 0.0005 |
|  | C | 0.1418 | 0.9899 | 0.0416 | 0.3418 |  | 0.7946 | 0.6071 | 0.0905 | 0.0885 |
|  | HC | 0.9994 | -0.0360 | 0.2671 | 0.0015 |  | 0.9828 | 0.1846 | 0.1782 | 0.0125 |
| **Microbial metabolites** | ACE | -0.8921 | 0.4519 | 0.0252 | 0.5072 |  | 0.6490 | 0.7608 | 0.0169 | 0.6552 |
|  | PRO | -0.9948 | 0.1016 | 0.0927 | 0.0760 |  | -0.1703 | 0.9854 | 0.0774 | 0.1274 |
|  | IBUT | -0.9679 | -0.2512 | 0.2298 | 0.0025 |  | -0.4397 | -0.8982 | 0.0519 | 0.2544 |
|  | BUT | -0.9286 | -0.3710 | 0.0559 | 0.2204 |  | -0.9966 | -0.0825 | 0.1880 | 0.0065 |
|  | IVAL | -0.9671 | -0.2545 | 0.3286 | 0.0005 |  | -0.8196 | -0.5729 | 0.0621 | 0.1974 |
|  | VAL | -0.9995 | 0.0311 | 0.0065 | 0.8446 |  | 0.0516 | -0.9987 | 0.0765 | 0.1384 |
|  | TVFA | -0.9861 | 0.1660 | 0.0450 | 0.2949 |  | 0.0128 | 0.9999 | 0.0137 | 0.7176 |
|  | ACE/PRO | 0.9761 | 0.2171 | 0.0727 | 0.1399 |  | 0.8097 | -0.5869 | 0.0898 | 0.0915 |
| **Blood metabolites** | GLU | -0.9962 | 0.0876 | 0.0488 | 0.2524 |  | 0.9995 | 0.0312 | 0.0276 | 0.5037 |
|  | LDH | 0.1434 | 0.9897 | 0.1776 | 0.0050 |  | 0.8397 | -0.5431 | 0.0613 | 0.1984 |
|  | CK | 0.2502 | 0.9682 | 0.1303 | 0.0315 |  | 0.9681 | -0.2508 | 0.0746 | 0.1529 |
|  | TG | 0.4901 | 0.8717 | 0.1400 | 0.0180 |  | 0.8153 | -0.5790 | 0.0462 | 0.3148 |
|  | LDL-C | 0.3913 | 0.9202 | 0.1090 | 0.0495 |  | 0.7978 | -0.6029 | 0.1188 | 0.0425 |
|  | HDL-C | 0.3496 | 0.9369 | 0.1371 | 0.0315 |  | 0.4871 | -0.8734 | 0.1611 | 0.0120 |
|  | TC | 0.3425 | 0.9395 | 0.1238 | 0.0405 |  | 0.6302 | -0.7764 | 0.1487 | 0.0145 |
|  | NEFA | -0.5631 | 0.8264 | 0.0037 | 0.9080 |  | -1.0000 | -0.0046 | 0.1275 | 0.0360 |
|  | FAS | -0.9633 | 0.2685 | 0.0129 | 0.7036 |  | 0.6163 | 0.7875 | 0.0081 | 0.8231 |
|  | LPL | 0.3325 | 0.9431 | 0.0557 | 0.2174 |  | -0.0509 | -0.9987 | 0.0504 | 0.2834 |
|  | HSL | 0.2003 | 0.9797 | 0.0539 | 0.2314 |  | 0.3262 | -0.9453 | 0.0637 | 0.2004 |

**Note:** RDA1 and RDA2 denote the correlation between environmental factors and the ranking axis, R^2^ denotes the determinant of environmental factors and species distribution, and the smaller the value, the smaller the effect of environmental factors on species distribution. P-value represents the significance test of the correlation.

Figures

**Fig. S1** Seasonal dynamics of environmental factors and forage nutrients in different grazing months. Based on scatter plot, locally weighted regression (LOESS) was used to show the dynamic changes of environmental factors such as monthly mean temperature (A: day; B: night) and monthly mean humidity (C) in different grazing months. Meanwhile, the nutrient composition of forages in different grazing months was shown based on scatter plot and LOESS analysis, such as forage biomass (D), dry matter (E), crude protein (F), crude fat (G), crude fiber (H), nitrogen-free invasion (I), neutral detergent fiber (J), acid detergent fiber (K), lignin (L), crude ash (M), cellulose (N) and hemicellulose (O). The levels of differences in environmental factors and forage nutrients between different grazing months were compared based on one-way ANOVA.

**Fig. S2.** Composition of rumen fungal communities in yaks and yellow cattle during different grazing months. (A) And (C) represent the stacking plots of rumen fungal phylum levels in yaks and yellow cattle during different grazing months. (B) And (D) represent the stacking plots of the levels of anaerobic fungi in the rumen of yaks and yellow cattle during different grazing months.

**Fig. S3** Effects of different grazing months on the diversity and structure of rumen AF community of yak and cattle. Based on scatter plots, the results show that Chao1 diversity indices of rumen AF of yak (A) and cattle (B) in different grazing months (Nov., Jan., Mar., May, July and Sept.) and seasons (cold and warm seasons). Violin plot shows the Chao1 and Shannon diversity indices of rumen AF community between yak (C) and cattle (D) in different grazing months. Friedman test was used to compare the diversity of rumen AF community Chao1 in different months while Friedman test was used to compare the diversity of rumen AF community in different grazing seasons and species. The PCoA based on Bray-Curtis distance (E) shows the effects of grazing months and seasons on rumen AF community structure in yak and cattle.

**Fig. S4** Composition of rumen AF community in yak and cattle during different grazing months. A: Upset plots shows the distribution of the AF OTUs in yak and cattle samples from different grazing months (Nov., Jan., Mar., May, July and Sept.). Pie charts show the common rumen AF community at genus level of yak and cattle in different grazing months, and the relative percentages of different rumen anaerobic fungal communities in inner and outer rings for yak and cattle, respectively. Percentage stacked bar graph show the distribution of rumen core AF communities in yak (B) and cattle (C) during different grazing months.

**Fig. S5** Effects of different grazing months on rumen anaerobic fungi in yak and cattle. Rumen AF were identified to be important for yak (A) and cattle (B) in different grazing months (Nov., Jan., Mar., May, July and Sept.) by LEfSe. The bar graphs show significant differences of AF in rumen between yak and cattle in different grazing months. Different colors represent different grazing months. MDS maps show the distribution of rumen AF of both (C), yak (D) and cattle (E) during cold and warm seasons, using the similarity matrix of the Random Forest model.

**Fig. S6** Pearson correlation between climatic variables, forage nutrients, and rumen and blood metabolites of yak collected in different grazing months. All data were standardized using the Z-score standardization method.

**Fig. S7** Pearson correlation between climatic variables, forage nutrients, and rumen and blood metabolites of cattle collected in different grazing months. All data were standardized using the Z-score standardization method.

**Fig. S8** Variation partitioning analysis of the relative contributions of grazing months, seasons, and Species to the variation in rumen AF community. (A) The explanatory power of different grazing months and seasons on the dynamic changes of rumen AF community in yak. (B) The explanatory power of different grazing months and seasons on the dynamic changes of rumen AF community in cattle. (C) Relative contribution of different grazing months, seasons and species to the variation of rumen AF community.

**Fig. S9** Beta−NTI distribution. The degree of community transformation was judged by beta-NTI (beta-NTI > 0, high turnover; beta-NTI > 0, low turnover). (A) Denotes the transformation degree of rumen AF community in yak during warm season (May, July and Sept.). (B) Denotes the transformation degree of rumen AF community in yak during cold season (Nov., Jan. and Mar.). (C) Denotes the transformation degree of rumen AF community in cattle during warm season. (D) Denotes the transformation degree of rumen AF community in cattle during cold season.

1. * **Corresponding author:**

   Ping Yan (PhD); E-mail: yanping@caas.cn

   Ghasem Hosseini Salekdeh (PhD); E-mail: h_salekdeh@abrii.ac.ir

   Xuezhi Ding (PhD); E-mail: dingxuezhi@caas.cn

   †These authors have contributed equally to this study. [↑](#footnote-ref-1)
